# Investigation of North Sea high-frequency sea-level extremes using long-term sea-level measurements

**DOI:** 10.1007/s11069-026-08343-y

**Published:** 2026-08-26

**Authors:** Mia Pupić Vurilj, Gozde Guney Dogan, Marijana Balić, José A. A. Antolínez, Jadranka Šepić

**Affiliations:** 1 Department of Hydraulic Engineering, Delft University of Technology, Delft, Netherlands; 2 Faculty of Science, University of Split, Split, Croatia; 3 University of Split, Split, Croatia

**Keywords:** High-frequency sea-level oscillations, Sea level extremes, North Sea, Clustering, Compound events

## Abstract

Extreme sea-level events occur across a range of temporal and spatial scales, including high-frequency oscillations (periods T < 2 h). However, most research uses hourly or daily data and neglects higher-frequency processes such as seiches and meteotsunamis. This study investigates the characteristics of high-frequency (HF) sea-level extremes along the North Sea coast. Long-term (1993–2025) measurements were analysed from 29 tide gauges. After quality control, the astronomical tide was removed to isolate residuals, which were decomposed into low-frequency (T > 2 h) and high-frequency (T < 2 h) components. Next, extremes were extracted from the HF signal alone (HF extremes) and from the residual (residual extremes). Clustering techniques (K-medoids with Dynamic-Time-Warping and Euclidean distances) were applied to HF extremes to classify event types and identify regional patterns. HF extremes were grouped into six event types, and selected stations yielded five spatial clusters. Each event type was characterised by period, intensity, and its relationship to residual extremes. HF extremes were generally low compared with tidal ranges, and compound events (i.e., events in which an HF extreme coincided with a residual extreme) were infrequent (12.2% of HF extremes on average). Nevertheless, during compound events, alignment of HF and residual peaks substantially amplified sea levels. Spatial patterns highlighted the most intense events along the Dutch coast (IJmuiden Buitenhaven, Scheveningen, Brouwershavensche Gat, and Terschelling). These results were influenced by differences in local environmental conditions and sampling. Therefore, this study advances understanding of HF sea-level variability and compound events along the North Sea coast, supporting improved coastal hazard assessments.

**Supplementary Information:**

The online version contains supplementary material available at https://doi.org/10.1007/s11069-026-08343-y.

## Introduction

Due to anthropogenic climate change, the global mean sea level is continuously rising. Together with changes in extreme weather, this rise increases both the frequency and magnitude of extreme sea-level events (Vitousek et al. 2017; Taherkhani et al. 2020). Events that were once rare, occurring perhaps once per century, are projected to become annual at over half of all tide-gauge sites by the end of the twenty-first century (IPCC 2021). This trend threatens densely populated coastal regions, including countries with sophisticated coastal-defence systems such as the Netherlands and the United Kingdom (Kron 2013). Consequently, traditional engineering designs that rely on historical variability may no longer be adequate. Contemporary engineering is therefore progressively incorporating probabilistic methods, machine learning, and coupled physical–statistical models to capture the effects of climate change (IPCC 2022; Pupić Vurilj et al. 2025; Bracco et al. 2025).

Extreme sea-level events are composed of several interacting components. These include the mean sea level, astronomical tide, and a range of mostly atmospherically induced processes. The atmospheric processes occur at various time scales, including planetary-scale periods (> 10 days), synoptic-scale periods (a couple of hours to 10 days), and shorter periods. Planetary-scale atmospheric processes drive long-term sea-level variations, while synoptic-scale processes generate storm surges and wave-driven setup. At even shorter periods, atmospheric forcings can generate long-period ocean waves, infragravity waves, and wind waves (Pugh and Woodworth 2014). The sea-level response to these processes is mostly governed by atmospheric pressure and wind stress, and is further modulated by local bathymetry. Conclusively, tidal forcing, background sea level, atmospheric circulation and local coastal geometry shape the observed sea-level extremes (Arns et al. 2020; Woodworth et al. 2019; Muis et al. 2023; Batstone et al. 2013).

In this study, sea-level oscillations with short periods ranging from a few minutes, as resolved by the measurement resolution, up to two hours are termed *high-frequency (HF) sea-level oscillations.* Their extremes are referred to as *high-frequency (HF) sea-level extremes*, or short *HF extremes*. These phenomena encompass various wave types, including long ocean waves, harbour seiches, edge waves, and infragravity waves. Among these, various types of tsunamis represent the strongest HF extremes; if they are generated by atmospheric processes, they are called meteorological tsunamis (meteotsunamis; Monserrat et al. 2006; Vilibić et al. 2025). These long waves may be generated by rapidly moving atmospheric pressure disturbances, squall lines, and frontal passages. Subsequently, they can be amplified through Proudman, Greenspan, or harbour resonance (Pugh and Woodworth 2014; Williams et al. 2021; Sibley et al. 2021; Monserrat et al. 2006; Pattiaratchi and Wijeratne 2015; Vilibić et al. 2025). Studies worldwide have shown that atmospherically generated HF extremes can contribute 5–60% to local extreme sea levels, and occasionally constitute the entire extreme, particularly in semi-enclosed basins such as the Mediterranean Sea, Baltic Sea, Yellow Sea, and Great Lakes (Heidarzadeh et al. 2025; Šepić et al. 2015; Vilibić and Šepić, 2017; Ruić et al. 2023; Heidarzadeh and Rabinovich 2021; Vilibić et al. 2025). Consequently, HF extremes can substantially influence total water levels and coastal currents (Rabinovich 2009; Vilibić and Šepić, 2017; Reiners et al. 2021; Heidarzadeh and Rabinovich 2021).

The North Sea’s semi-enclosed configuration, shallow bathymetry, and exposure to strong meteorological forcing create favourable conditions for both residual and HF extremes. Residual extremes (often interchangeably referred to as storm surges) have been studied extensively in the North Sea. For example, numerous studies have examined storm surge magnitudes (e.g., Dangendorf et al. 2016; Ganske et al. 2018; de Valk and van den Brink, 2023). Other studies have highlighted the importance of their temporal characteristics: duration, multi-peak behaviour, and clustering (Haigh et al. 2016; Stephens et al. 2020; Anderson et al. 2019; Jenkins et al. 2023; Pupić Vurilj et al. 2025). Historical events, such as the 1953 North Sea Flood, remain stark reminders of this region's vulnerability to residual extremes (Baxter 2005; Gerritsen 2005).

HF extremes remain comparatively less explored. However, studies have identified meteotsunamis and seiches in the North Sea that were triggered by mesoscale weather systems, such as cold fronts and convective cells. (Williams et al. 2021; de Jong et al. 2003, 2021; Sibley et al. 2021). For example, in Dutch North Sea ports, long ocean waves are known to generate standing waves, or harbour seiches (de Jong et al. 2003; de Jong and Battjes 2004). Seiches can increase hydrostatic loads and thus cause damage to coastal defence structures (de Jong et al. 2021). The associated strong currents also influence wave propagation and may contribute to the dynamic loading of coastal defences.

To address these risks, de Jong et al. (2021) applied bivariate probability distributions to the ports of Rotterdam, IJmuiden, and Terneuzen to determine the required allowance for the Net Seiche Effect (NSE), defined as the water-level increase caused by HF oscillations. Their methodology produced specific allowances that are now used in the design and safety assessment of primary flood defences and sea locks in these three ports. The study also showed that the winter meteotsunami events are the primary concern for coastal defence assessments, as they may coincide with the large north-westerly storm surges that drive extreme sea levels along the Dutch North Sea coast. Summer events, meanwhile, may be relevant for different reasons, as warmer months bring more people to near-coastal daily and recreational activities (e.g., Sibley et al. 2021). This highlights the complexity of HF extremes and their impacts.

More recently, Heidarzadeh et al. (2025) documented a 17–23 cm meteotsunami that coincided with a long‑duration storm surge in the UK in 2023. Such instances underscore the need to identify and characterise HF extremes to understand how, together with coincident storm surges, they may exacerbate flooding hazards under a changing climate. Currently, however, most studies in the North Sea are limited in temporal or spatial extent, and comprehensive analyses of combined storm surge and HF effects remain scarce (e.g., Williams et al. 2021; de Jong et al. 2021). Therefore, further investigation in the region is required.

The present study aims to enhance the understanding of HF sea-level extremes along the North Sea and their co-occurrence with storm surges. To achieve this, sea-level measurements from a dense, long-term network of 29 tide gauges, spanning the period 1993–2025, are analysed using clustering techniques. The analysis identifies both standalone HF extremes and compound events, i.e., events in which a HF extreme coincides with a residual extreme (storm surge). By examining how these processes coincide, this study presents a step towards more accurate assessments and anticipation of coastal flood risk.

The study begins with a detailed outline of the materials and methods used to define the HF extremes, followed by spatial and event-type clustering to analyse their variability. The Results provide a characterisation of these spatial clusters and event types, including their relationship with residual extremes. The findings are then interpreted in the Discussion chapter, highlighting their relevance and limitations. The study concludes by summarising key findings and offering recommendations for future research.

## Materials and methods

**Fig. 1:**
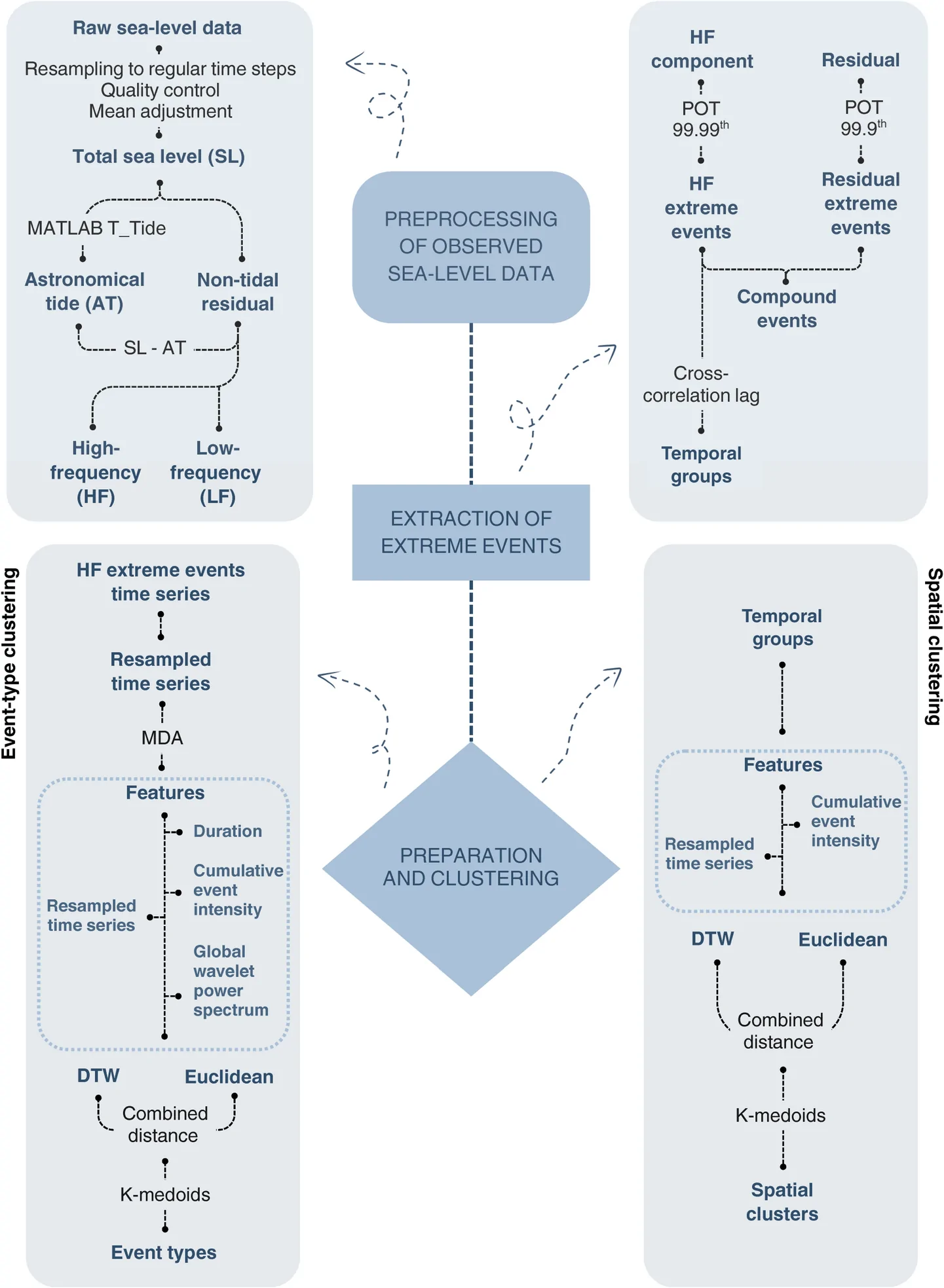
Flowchart illustrating the methodology of the study, detailing three key stages: (I) preprocessing of observed sea-level data, (II) extraction of extreme events, (III) preparation and clustering

As shown in Fig. 1, the methodology of this study consists of three main stages, each represented in more detail in a separate subchapter. The three steps are summarised as follows:

I. *Preprocessing of observed sea-level data*: Sea-level data were collected from 29 tide gauge stations across the North Sea and subjected to a robust quality-control procedure. Following de-tiding, the residual sea-level series were decomposed into low-frequency (*LF*; T > 2 h) and high-frequency (*HF*; T < 2 h) components.
II. *Extraction of extreme events*: Using the Peak-Over-Threshold (POT) method, extremes were extracted from two types of time series: (i) the high-frequency signal alone (*HF extremes*), and (ii) the (full) residual signal (*residual extremes*). Compound events were defined as events in which an HF extreme coincided in time with a residual extreme.
III. *Preparation and clustering*: Clustering techniques were applied to HF extreme events to classify event types and identify coherent regional patterns along the North Sea coast. This step encompasses preprocessing and clustering procedures for (i) spatial clustering and (ii) event types, treated separately.

### Preprocessing of observed sea-level data

**Fig. 2:**
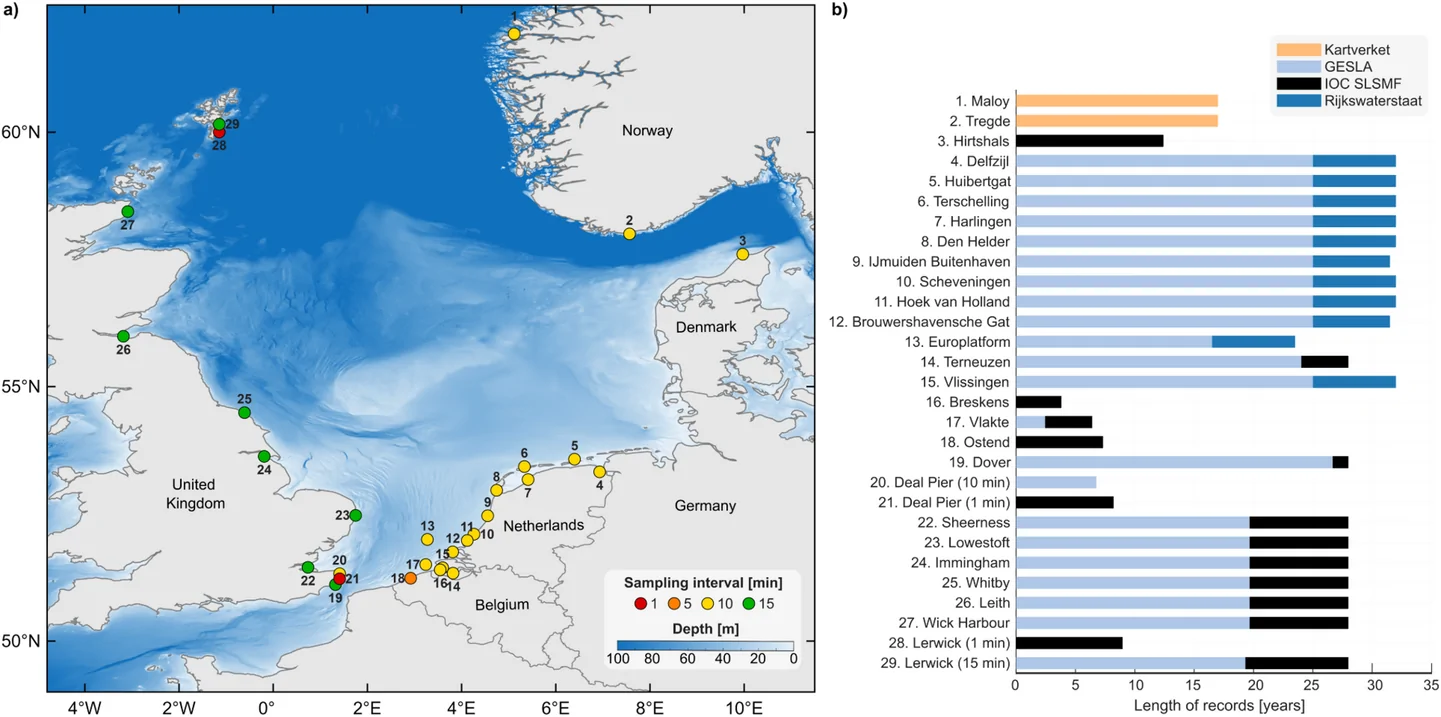
**a** Map of the North Sea station locations. The sampling interval (in minutes) is represented by the circle colour. Station names and metadata are listed in Table 1 and Table S1 in Supplementary Information. Note that stations 20 and 28 have been slightly shifted from their actual geographic locations to enhance map clarity. **b** Length of the records per station. The stacked bars indicate the data source combinations used for each station’s record: IOC SLSMF, GESLA, Rijkswaterstaat and Kartverket (see legend for colour coding)

Time series of sea-level measurements from 29 stations across the North Sea (Table 1, Fig. 2a), recorded at intervals between 1 and 15 min, were obtained from the following sources: (i) the IOC Sea Level Station Monitoring Facility (SLSMF, https://www.ioc-sealevelmonitoring.org/); (ii) the Global Extreme Sea Level Analysis dataset (GESLA, https://www.gesla.org/, Haigh et al. 2022); (iii) Rijkswaterstaat Waterinfo (RWS, https://waterinfo.rws.nl/thema/Waterbeheer), the official Dutch authority for water management; (iv) Kartverket (NHS, https://vannstand.kartverket.no/tideapi_en.html), the Norwegian Mapping Authority Hydrographic Service. Where possible, data from multiple sources at the same location were combined (Fig. 2b) to produce the longest, highest-quality continuous records (see Table S1 in Supplementary Information for details). The quality of the downloaded time series depended on whether, and to what extent, quality control had been applied by the provider prior to making these data publicly available. Here, quality control refers to the identification of spikes, shifts, drifts, and other data acquisition and processing errors. For example, IOC data are not subjected to any quality control procedure, whereas Kartverket data undergo quality control prior to release.

**Table 1:** Station information and statistical thresholds. Includes observation periods (start/end dates), local environmental setting, the 99.9th percentile of the residual signal, and the 99.99th percentile of the HF signal

Station		Data start time	Data end time	Setting	99.9th perc. of the residual [cm]	99.99th perc. of the HF signal [cm]
1	*Maloy*	1.1.2008 0:00	1.1.2025 0:00	Harbour in a channel	61.83	3.42
2	*Tregde*	1.1.2008 0:00	1.1.2025 0:00	Archipelago	61.19	1.96
3	*Hirtshals*	3.8.2008 12:20	1.1.2021 0:00	Open coast, close to a harbour	86.39	12.7
4	Delfzijl*	1.1.1993 0:00	31.12.2024 23:00	Harbour	197.40	14.75
5	Huibertgat*	1.1.1993 0:00	31.12.2024 23:00	Open coast, between islands	149.19	10.55
6	Terschelling*	1.1.1993 0:00	31.12.2024 23:00	Open coast	129.14	16.68
7	Harlingen*	1.1.1993 0:00	31.12.2024 23:00	Harbour	183.91	7.46
8	Den Helder*	1.1.1993 0:00	31.12.2024 23:00	Wide channel	142.27	10.02
9	IJmuiden Buitenhaven*	1.1.1993 0:00	30.6.2024 22:50	Harbour	139.18	20.05
10	Scheveningen*	1.1.1993 0:00	31.12.2024 23:00	Harbour	136.18	18.86
11	Hoek van Holland*	1.1.1993 0:00	31.12.2024 23:00	Harbour	129.16	10.92
12	Brouwershavensche Gat*	1.1.1993 0:00	30.6.2024 22:50	Open coast, sandbar	144.77	20.66
13	Europlatform*	30.6.2001 23:00	31.12.2024 23:00	Offshore	111.71	10.49
14	Terneuzen*	1.1.1993 0:00	1.1.2021 0:00	Harbour in an estuary	140.37	13.56
15	Vlissingen*	1.1.1993 0:00	31.12.2024 23:00	Small harbour in an estuary	136.05	10.52
16	*Breskens*	9.3.2017 9:10	1.1.2021 0:00	Small harbour in an estuary	111.50	14.01
17	*Vlakte*	4.8.2014 0:00	1.1.2021 0:00	Offshore, at estuary mouth	107.54	8.76
18	*Ostend*	6.9.2013 0:00	1.1.2021 0:00	Harbour	120.48	17.19
19	Dover*	1.1.1993 0:00	1.1.2021 0:00	Open coast	85.96	6.71
20	*Deal Pier (10 min)*	1.1.2006 0:10	05.10.2012 23:50	Open coast	98.25	8.16
21	*Deal Pier (1 min)*	5.10.2012 9:09	21.12.2020 21:27		95.79	9.28
22	*Sheerness*	1.1.1993 0:15	1.1.2021 0:00	Estuary	131.72	11.97
23	Lowestoft*	1.1.1993 0:15	1.1.2021 0:00	Open coast, in front of a harbour	113.84	9.94
24	Immingham*	1.1.1993 0:15	1.1.2021 0:00	Harbour in an estuary	102.58	5.36
25	Whitby*	1.1.1993 0:00	1.1.2021 0:00	Harbour in an estuary	82.86	5.42
26	*Leith*	1.1.1993 0:00	1.1.2021 0:00	Bay	92.88	8.87
27	Wick Harbour*	1.1.1993 0:15	1.1.2021 0:00	Harbour	70.29	6.86
28	*Lerwick (1 min)*	6.3.2009 17:31	24.2.2018 19:32	Harbour in a channel	53.59	21.21
29	*Lerwick (15 min)*	1.1.1993 0:00	1.1.2021 0:00		62.79	9.80

Stations used in spatial clustering are marked with an asterisk (*)

*Stations used in spatial clustering

Hence, all raw time series obtained from the sources above were subjected to the same preprocessing steps, consistent with the protocol described in Balić and Šepić's SHELDA dataset (2025, in review; https://doi.org/10.5194/essd-2025-767). These steps included: (i) resampling to regular time step intervals at a given location (time step to which series were resampled is equal to time step of measurements at each location; Fig. 2a); (ii) automatic removal of non-physical spikes and outliers; (iii) mean adjustment by subtracting the average value from each time series; and (iv) visual inspection within a ten-day window, followed by manual removal of spurious parts of the records. Subsequently, the tidal signal was estimated using the MATLAB package T_Tide (Pawlowicz et al. 2002), and at each station, significant tidal components were removed. The tidal component was considered significant if the signal-to-noise ratio (amplitude of a constituent over its uncertainty) was above 2. Next, residual series were obtained by subtracting the tidal signal from the original records. The residual time series were then filtered with a 2‑hour Kaiser–Bessel window, resulting in high-frequency (HF, T < 2 h) and low-frequency (LF, T > 2 h) series (e.g., Thomson and Emery 2024; Williams et al. 2021).

The obtained dataset included two tide gauges for each of two UK sites, Lerwick and Deal Pier. Lerwick has a 15-min gauge and a 1-min gauge. The latter record contained many gaps. At Deal Pier, there is a 10-min tide gauge and a 1-min tide gauge. Data from all these stations were used to maximise record coverage. Thus, 29 tide gauges were used for 27 locations, with varying sampling intervals and record lengths. Moreover, the dataset comprises stations situated in a range of environmental settings, illustrated in the Supplementary Information and noted in Table 1.

While 1-min sampling might be considered the optimal resolution for analysing HF oscillations and their location-specific characteristics, data at this resolution were unavailable for the majority of stations in this study. Nevertheless, previous research has successfully utilised coarser intervals for HF climatology. For instance, Ozsoy et al. (2016) constructed a climatology of relatively HF waves (3–5 h periods) in the UK using 15-min sampling intervals, and Williams et al. (2021) employed similar intervals to develop a meteotsunami climatology across Northwest Europe (2010–2017). Therefore, the use of 10- and 15-min tide-gauge data was deemed appropriate for this study. However, it is important to acknowledge that due to the sampling interval, some HF processes could not be captured. The derived HF series are thus dominated by longer-period phenomena, such as local seiches and long ocean waves (T > 20–30 min for most stations). Longer-period infragravity waves (T > 2 min) were only marginally captured at two specific sites: Deal Pier (1 min) and Lerwick (1 min).

### Extraction of extreme events

Using the Peak-Over-Threshold (POT) method, extremes were extracted from two separate time series: (i) the high-frequency signal alone (*HF extremes*), and (ii) the (full) residual (*residual extremes*).

**Fig. 3:**
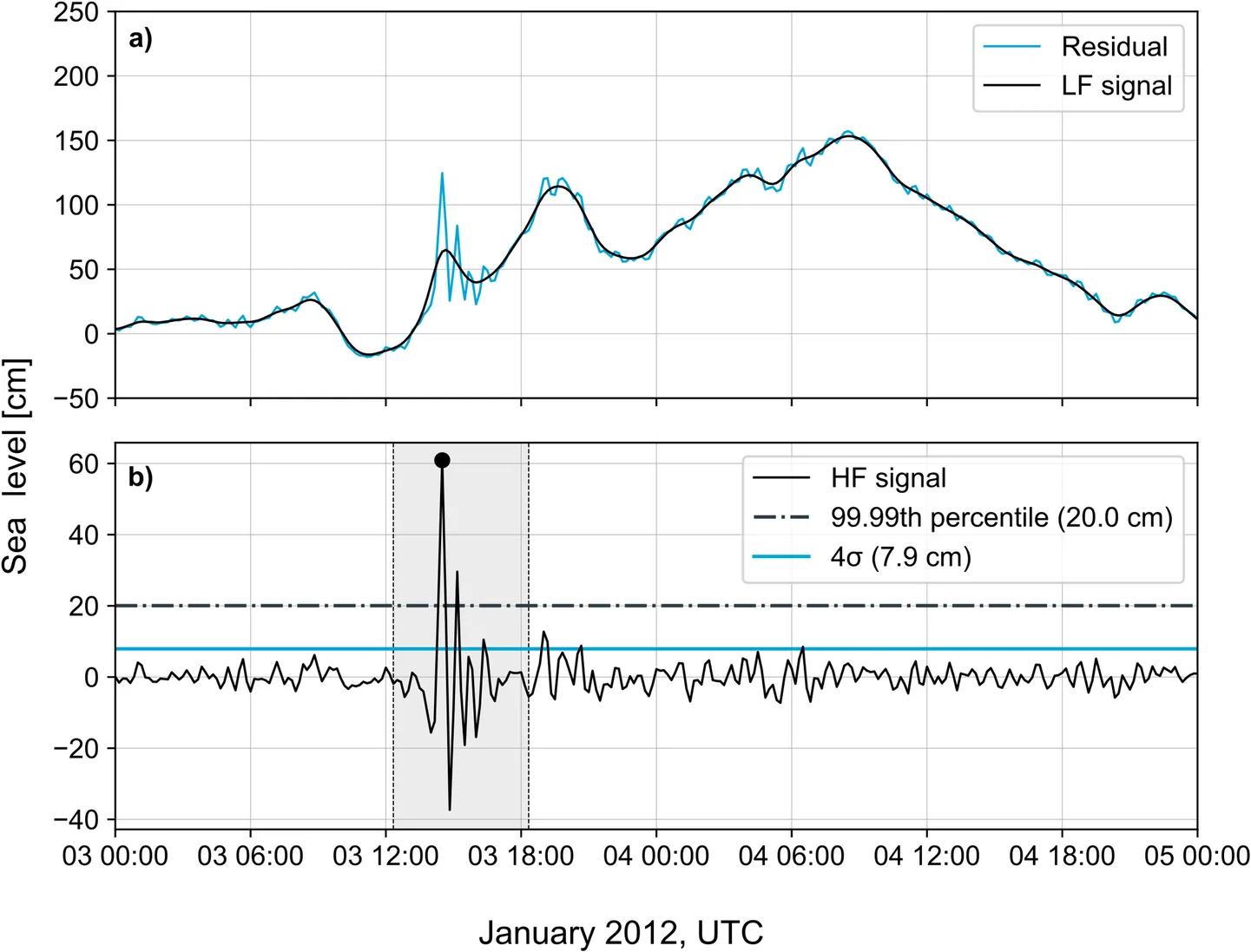
Example of an extreme event at IJmuiden Buitenhaven (station no. 9): **a** residual and low-pass filtered series (LF signal); **b** high-pass filtered series (HF signal). The dash-dot dark blue line marks the 99.99th-percentile threshold, while the solid blue line marks the 4 times the standard deviation threshold. The shaded area indicates the HF event duration, and the black dot marks the event maximum

HF extremes were defined as instances in which the HF series exceeded the station-specific 99.99th percentile (values in Table 1). To delineate an event, a 3-day independence criterion was applied. Event duration was defined as the continuous time interval surrounding each independent extreme during which the HF signal did not drop below 4 standard deviations for more than two consecutive hours. Operationally, following identification of an independent extreme, the algorithm expanded the event window both backward and forward in time until it detected at least two hours with HF amplitude below 4 standard deviations of the station's HF values (see Fig. 3).

In order to assess compound events involving storm surges and HF extremes, extremes from the residual series were extracted as well. Residual extremes were defined as instances where the residual series exceeded its station-specific 99.9th percentile (values in Table 1). Since storm surges are the dominant driver of coastal flooding in the North Sea, capturing a broader range of surge magnitudes is important to assess coastal risk. Therefore, a lower threshold (99.9th percentile) is applied to the residual series. Conversely, a stricter threshold (99.99th percentile) is necessary to isolate only the most extreme HF oscillations that pose a genuine risk. The choice of thresholds was motivated by the sample size as well, yielding ~ 2 events per year across stations for residual extremes and ~ 3 events per year for HF extremes. This is in line with previous studies (e.g., Haigh et al. 2016; Ruić et al. 2023). Because sampling intervals and record lengths differed among stations, the number of events varied as well, as shown in the Results chapter.

Compound events were identified by checking for residual extremes that occurred during an HF extreme. If multiple residual peaks were detected within a single HF event window, the peak with the highest amplitude was selected as the representative residual extreme. For each identified compound event, the contribution of the constituent LF and HF signals was calculated at the time of the residual extreme ($t_{res}$). In other words, the residual amplitude $R\left( {t_{res} } \right)$ is the sum of the HF and LF components at that instant (adopted from Ruić et al. 2023):

1 $$ \begin{array}{*{20}c} {R\left( {t_{res} } \right) = HF\left( {t_{res} } \right) + LF\left( {t_{res} } \right)} \\ \end{array} $$

The relative contribution of each component was then reported as the instantaneous value (in centimetres) or a percentage of the total residual amplitude (i.e., $\frac{{HF\left( {t_{res} } \right)}}{{R\left( {t_{res} } \right)}}$ and $\frac{{LF\left( {t_{res} } \right)}}{{R\left( {t_{res} } \right)}}$).

Here, it is important to note that the temporal resolution of the data affects how accurately HF extremes are captured. For example, along the Dutch North Sea coast, the main seiche oscillation has a known period of approximately 10–30 min (Veldman 2000; de Jong and Battjes 2004). Most of the observational records in this study are sampled at 10-min intervals, with some even at 15 min. At this resolution, the 30-min oscillation is only partially resolved, and shorter periods are not captured. As a result, the recorded HF maximum amplitudes likely underestimate the true peaks of these HF oscillations. This should be kept in mind when interpreting the magnitude of the HF extremes.

### Preparation and clustering

In this study, spatial (S) and event-type (E) clustering were implemented to examine the spatial and temporal characteristics of HF extremes along the North Sea coast.

First, spatial clustering was applied to identify characteristic regional patterns of HF extremes. This was based on the similarity of the time series of HF extreme events. It should be noted here that HF sea levels can reach potentially dangerous heights across diverse coastal settings, ranging from open coastlines (e.g., Daytona Beach, Florida, Sallenger et al. 1995 and Rabinovich 2020; Odesa, Black Sea, Šepić et al. 2018) to bays with strong resonant properties (e.g., Nagasaki Bay, Hibiya and Kajiura 1982; Port of Rotterdam, Netherlands, de Jong et al. 2021). For example, in 2017, a meteotsunami hit the English Channel and the North Sea, and HF extremes were recorded at tide gauges in both bays and open coast locations (Sibley et al. 2021; de Jong et al. 2021). Nonetheless, stations within harbours are generally more likely to exhibit amplified responses due to shoaling, narrowing and harbour resonance effects (e.g., Rabinovich 2009) than stations along the open coast. Thus, we expect the spatial clustering to, in addition to extracting characteristic regional behaviour, also group the stations of the same type (e.g., harbour vs. open coast) into similar clusters. This also serves as a test of our clustering method.

Complementing this spatial analysis, event-type clustering was performed across stations to identify recurring time series patterns in extreme HF oscillations. Station information was not included as a classification feature in event-type clustering. This way, the classification captured whether similar patterns emerge across diverse settings, and also whether the same physical event produces distinct patterns depending on the site-specific environment.

Commonly, extreme sea-level events are parametrised prior to clustering (Camus et al. 2021, 2024; Rueda et al. 2017). For example, Camus et al. (2024) classified extreme surge events using only maximum surge values, while Rueda et al. (2017) defined six storm attributes for regional pattern identification. In contrast, this study extends the framework of Pupić Vurilj et al. (2025, 2026) to cluster the time series of HF extremes, capturing both static and time-varying patterns, as explained in the following subchapters. To perform spatial clustering, it was necessary to use datasets with overlapping observation periods across all stations. Therefore, 17 stations with the longest and most complete records were selected for spatial clustering (marked in Table 1). In contrast, event-type clustering included all stations, regardless of record length or completeness. Since stations covered different time spans and the Maximum Dissimilarity Algorithm (explained below) influenced which events were retained, a degree of inhomogeneity was introduced into the event-type clustering results. Not all stations or years contributed equally to the analysis. Nevertheless, the clustering captured the principal types of HF extreme events recorded in the North Sea and offered insights into their spatial and temporal variability, and contribution to residual extremes.

A detailed overview of all steps, along with the distinction between spatial and event-type clustering, is provided in the following subsections and schematised earlier in Fig. 1.

#### Spatial clustering

In this section, the clustering of stations to identify regional patterns and distinct station types is described. The following steps were undertaken for preprocessing and clustering:

S I. *Cross-correlation lag*: Cross-correlation was applied to the HF series to quantify temporal lags between the selected 17 stations.
S II. *Event grouping*: HF events from all stations were grouped by aligning them based on their inter-station cross-correlation lags. Events occurring within this alignment window were considered part of the same *temporal group*. Therefore, a temporal group refers to a time interval initiated by an HF extreme event at least at one station. During grouping, a tolerance of ± 12.5 h was allowed to account for potential phase changes during extreme events that arise from tidal interactions. The tidal phase can be affected by depth changes due to elevated sea levels, and vice versa. For each temporal group, start and end times were determined by the earliest and latest detections across all stations. Additionally, if two events occurred less than 12.5 h apart, they were further merged into a single event.
  Additionally, the 99.99th-percentile maxima were extracted for each temporal group at every station (at which the event occurred), and the median time lag of these maxima between stations was calculated. These lags were not used for clustering, but later to assess event behaviour. For improved visualisation, these lags were interpolated using a Radial Basis Function (RBF) thin plate spline interpolation (Duchon 1976; Franke 1982).
S III. *Time series extraction*: For each identified temporal group, HF time series were extracted from every station in the network over the shared temporal interval. This shared interval was defined by the globally detected HF extreme event start and end times. Therefore, this extraction included series from all stations even if they did not individually record an HF extreme (i.e., did not cross the trigger threshold) within that window. This allowed the clustering to identify which stations experience extremes together, capturing regional variability in event occurrence.
S IV. *Temporal resampling*: Distance-based clustering methods require all data points to have the same number of features. Therefore, time series were resampled to a uniform number of time steps across all temporal groups, using the event with the largest time step and duration combination as the reference. As a result, all time series were resampled to 239 equidistant steps using linear interpolation. Since event durations differ, the resulting time step was event-specific. For example, a temporal group lasting 10 h had an interpolated time step of $\frac{10 h}{{239 steps}} = 0.04 h = 2.4 min$, while one lasting 40 h had a step of $\frac{40 h}{{239 steps}} = 0.17 h = 10.2 min$. The interpolated time step had a mean of 2.3 min and a maximum of 15 min. This resampling can affect event magnitudes by smoothing out some peaks and missing the moment of the maximum value. Thus, cumulative event intensity is included as an additional feature, as described in the next step. More details and an example are provided in the Supplementary Information.
S V *Features augmentation*: The cumulative intensity (*I*) per station per temporal group was added as an additional feature to the clustering process. It was defined as an integral of original (non-resampled) HF values that exceeded the 99.99th percentile over the temporal group’s duration (calculated in meter-hours (mh) but reported throughout the manuscript in centimetre-hours (cmh); Pupić Vurilj et al. 2025):
  2 $$ \begin{array}{*{20}c} {I = \mathop \smallint \limits_{{t_{i} }}^{{t_{f} }} HF_{above 99.99} \left( t \right) d} \\ \end{array} $$
  The feature set for clustering thus comprised a time-varying feature: (i) the resampled time series, i.e. shape (239 values per station per temporal group); and a static feature: (ii) the cumulative intensity (per station per temporal group). *Note*: Duration was not considered in spatial clustering, as we defined temporal groups (S III). Therefore, we are already comparing stations based on time series of the same duration. A schematic of the features is provided in the Supplementary Information.
S VI. *Distance matrices*: To enable clustering, distance matrices that quantify the pairwise dissimilarities between all data points (i.e., resampled time series or intensity values) were calculated. A distance matrix is a square table where each cell $\left( {i{,}j} \right)$ contains a numerical value representing the degree of dissimilarity between items * i* and *j*; lower values indicate high similarity, while higher values indicate distinct characteristics. Two specific metrics were employed:
  *Dynamic Time Warping (DTW)*: Pairwise dissimilarities between stations’ resampled HF time series were quantified using DTW, which accounts for temporal shifts by non-linear alignment of the time axis (Berndt et al., 1994). This enables the algorithm to locally stretch or compress sections of the time series to find the optimal match between two wave patterns, thereby accommodating differences in event propagation speed. When two time series are too dissimilar to be meaningfully aligned, the resulting DTW distance reflects this lack of similarity.
  *Euclidean*: The dissimilarities in intensity were quantified using Euclidean distance. This metric calculates the straight-line geometric distance between two intensity vectors in a multi-dimensional space (Gower, 1982).
  S VII. *Combined distance*: Both DTW and Euclidean distance matrices were independently scaled to [0, 1] using min–max normalisation. The matrices were then combined with equal weighting, following the approach of Bellanger et al. (2021):
    3 $$ \begin{array}{*{20}c} {D = \alpha D_{DTW} + \beta D_{Euclidean} } \\ {\alpha + \beta = 1} \\ \end{array} $$
    where $\alpha = \beta = 0.5$ are fixed parameters weighting the contribution of each data source to the dissimilarity matrix *D*, $D_{DTW}$ is the DTW dissimilarity matrix, and $D_{Euclidean}$ is the Euclidean matrix.
  SVIII. *K-medoids*: Finally, the stations were clustered using the precomputed dissimilarity matrix *D**,* with the K-medoids algorithm (Kaufman et al. 1990), initialised with K-medoids +  + (adapted from K-means +  + ; ; Arthur and Vassilvitskii 2007). The K-medoids algorithm was selected because it can handle any dissimilarity matrix and is based solely on inter-observation distances. The reader is referred to the Supplementary Information for the definition of the algorithm. The optimal number of clusters was determined from the silhouette score and cluster stability score (Rousseeuw 1987), resulting in 5 spatial clusters, presented in the Results chapter. The score is presented in the Supplementary Information.

#### Event types

In this section, the clustering of HF extreme events to identify distinct event types is described. The following steps were undertaken for preprocessing and clustering (repeated steps are not described in detail):

E I. *Temporal resampling*: All time series at each station were resampled to 313 equidistant time steps using linear interpolation (see **S IV** and Supplementary Information). The interpolated time step had a mean of 1.4 min and a maximum of 10 min. The number of steps differs from that in spatial clustering because the two clustering procedures used different reference events, as the input data varied.
E II. *Maximum Dissimilarity Sampling*: Clustering was carried out only on a representative subset to prevent biases caused by present uneven sample densities. The subset was selected using the Maximum Dissimilarity Algorithm (MDA), which identifies a specified number of data points (i.e., time series) from a larger dataset such that the chosen points are maximally dissimilar to each other. This ensured adequate representation of less frequent event types in the clustering process (Camus et al. 2011; Athanasiou et al. 2021; Scott et al. 2020). The reader is referred to the Supplementary Information for further definition of the algorithm.
  A subset of 55% of the total number of events was chosen. This decision was motivated by the need for broader spatial coverage across the basin. Using a subset smaller than 55% resulted in the exclusion of locations such as Maloy, thereby omitting the entire northeastern coastal region from the analysis. It should be noted, however, that Tregde (another Norwegian station) was omitted from the final subset, as its inclusion would require a subset of 87% of all events.
E III. *Features augmentation*: For spatial clustering, time series were extracted based on temporal groups, ensuring that stations were compared based on time series of equal duration. As a result, no compensation for the event duration was necessary. In contrast, event-type clustering directly compares the shapes of long- and short-duration events. In this context, “shape” describes the relative evolution of the HF signal over time, focusing on the pattern of fluctuations rather than event length. This is why duration was added as an equally weighted feature in event-type clustering. Cumulative event intensity and global wavelet power spectrum of each event were included as secondary features.
  The global wavelet power spectrum was calculated for each event to distinguish events based on their dominant periods, helping to separate different hydrological phenomena. The continuous wavelet transforms (CWT) were computed using the complex Morlet wavelet (PyWavelet Python package with `cmor1.5–1.0` parameter; Lee et al. 2019; Morlet et al. 1982). Each time series was first resampled to 15-min intervals. The CWT was then applied over 16 logarithmically spaced scales, with a sampling period of 15 min, to obtain the wavelet power spectrum (up to a period of 240 min). Finally, the global wavelet power spectrum was derived by averaging the power across time for each series (Torrence and Compo 1998). While this process removes temporal information, which is often the main advantage of wavelet over Fourier analysis, the global wavelet spectrum was selected over the traditional Fourier spectrum because it is better suited for transient, non-stationary time series. Through this approach, a spectral signature for each event was obtained, enabling efficient comparison of dominant periods to distinguish between possible different hydrological phenomena.
  The feature set for clustering thus comprised a time-varying feature: (i) the resampled time series, i.e. shape (313 values per event); as well as static features: (ii) the event duration, (iii) the cumulative event intensity, and (iv) the global wavelet power spectrum of the event. A schematic of the features is provided in the Supplementary Information.
E IV. *Distance matrices*:
  *Dynamic Time Warping (DTW)*: Applied to the resampled HF event time series.
  *Euclidean distance*: Applied separately to event durations, cumulative intensities, and global wavelet power spectrum.
E V. *Combined distance*: Each distance matrix was normalised to [0, 1] using min–max scaling. A composite dissimilarity matrix was then constructed as:
  4 $$ \begin{gathered} D = \alpha D_{{DTW}} ~ + ~\beta D_{{Euclidean\_Duration}} + \gamma D_{{Euclidean\_Intensity}} + \delta D_{{Euclidean\_GlobalPower}} \hfill \\ \alpha + ~\beta ~ + ~\gamma ~ + ~\delta = 1 \hfill \\ \alpha = 0.3,~~\beta = 0.3,~~\gamma = 0.2,~~\delta = 0.2~~ \hfill \\ \end{gathered} $$
  where $\alpha , \beta ,\gamma , \delta \in \left[ {0,1} \right]$ are fixed parameters weighting the contribution of each data source to the combined dissimilarity matrix *D*; $D_{DTW}$ is the DTW dissimilarity matrix; and $D_{Euclidean\_Duration} , D_{Euclidean\_Intensity} ,$ and $D_{Euclidean\_GlobalPower}$ are the Euclidean matrices of the duration, cumulative intensity, and global wavelet power spectrum features, respectively.
  To construct the composite dissimilarity matrix, equal weights were assigned to the time series shape (*α*) and event duration (*β )*, together accounting for 60% of the total weight. This balanced weighting was introduced to compensate for the information loss of event duration caused by the temporal resampling of the data. The remaining 40% of the weight was distributed equally between event intensity (*γ*) and the global wavelet power spectrum (*δ*). These features serve as secondary classifiers without allowing them to dominate the clustering driven by the primary temporal dynamics. The weight distribution was guided by a sensitivity analysis (not presented here for brevity).
E VI. *K-medoids*: Event types were clustered using the precomputed dissimilarity matrix *D,* with the K-medoids algorithm, initialised with the K-medoids +  + algorithm. The optimal number of clusters was determined from the silhouette score, resulting in 6 event types, presented in the Results chapter. The score is presented in the Supplementary Information.

For more methodological details, see the Supplementary Information.

## Results

### HF extreme events

**Fig. 4:**
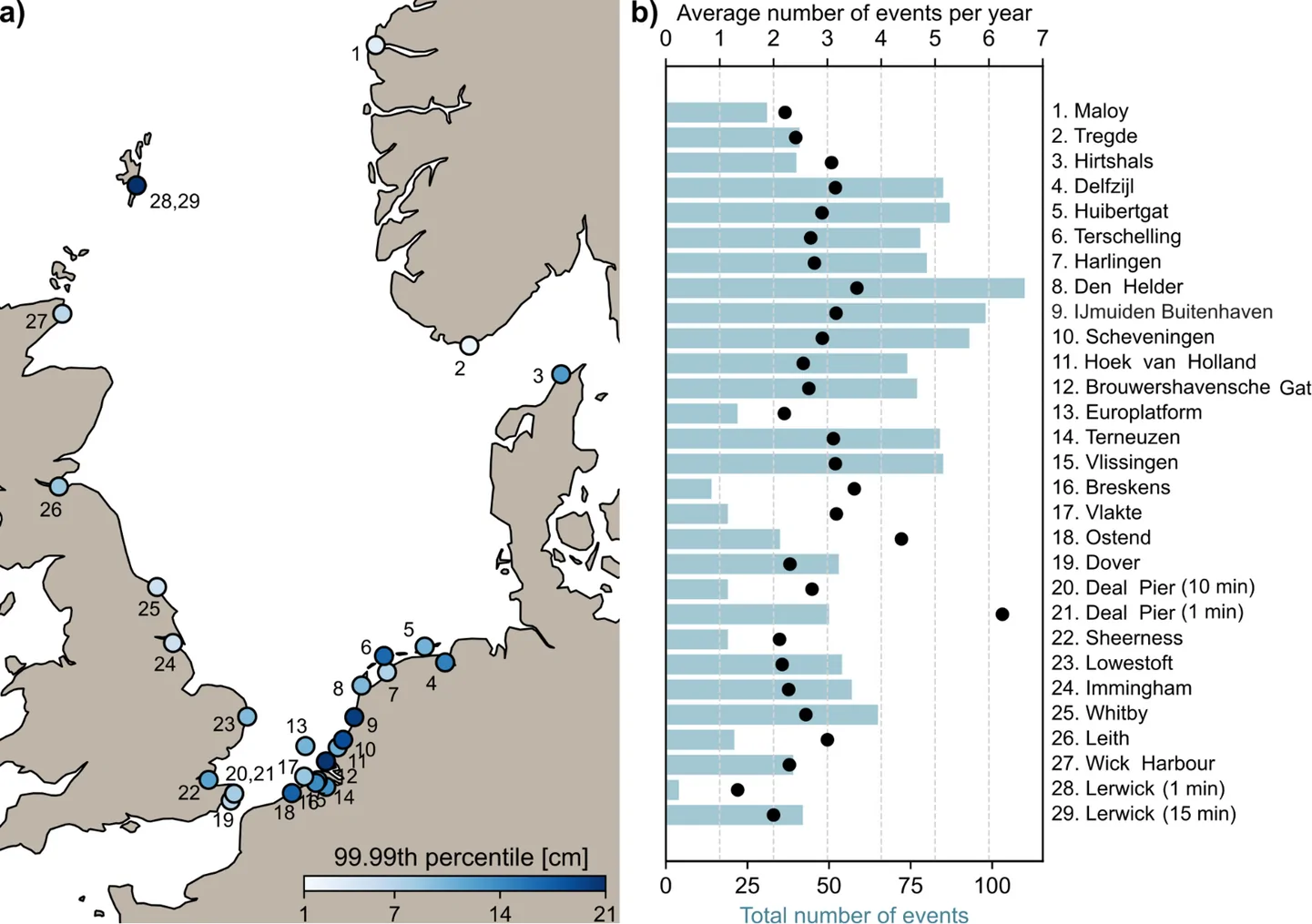
**a** 99.99th-percentile threshold at each station in cm, and **b** Total number of events (bars; biased by record length) and the average annual number of events (dots) per station

The value (in cm) of the 99.99th-percentile threshold used for extracting HF extremes varied substantially along the North Sea coast, ranging from 2 cm (at Tregde) to 21 cm (at Lerwick 1-min tide gauge). In total, 1,550 HF extreme events were identified, corresponding to an average of ~ 3 events per station per year. Given that the total counts are affected by uneven record lengths and gaps, the annual frequency is a more reliable measure of variability. All are shown in Fig. 4.

The variations in the strength of events and their number per year are consistent with physical drivers acting at local to regional scales, further explored in the Discussion chapter. Beyond physical processes, variability may also arise from differences in sampling intervals between stations: stations with shorter sampling intervals tend to have higher thresholds. For example, at Lerwick, the 15-min tide gauge had a 10 cm threshold, while the 1‑min tide gauge had a 21 cm threshold. This is in line with a well-known fact that a longer sampling time step significantly underestimates heights and return periods of extreme sea levels (e.g., Tsimplis et al. 2009). At Deal Pier, nonetheless, this difference was smaller: the 10-min tide gauge at Deal Pier had a threshold of 8 cm, and the corresponding 1-min tide gauge had a threshold of 9 cm. This suggests that there were likely no significant sea level oscillations at periods shorter than 10 min at Deal Pier.

Station Tregde exhibited a very low 99.99th-percentile threshold of only 2 cm, indicating that virtually no HF oscillations were recorded at this site. This might be due to the station’s unique local setting within an archipelago, or the relatively long sampling interval of 10 min.

### Spatial clusters

**Fig. 5:**
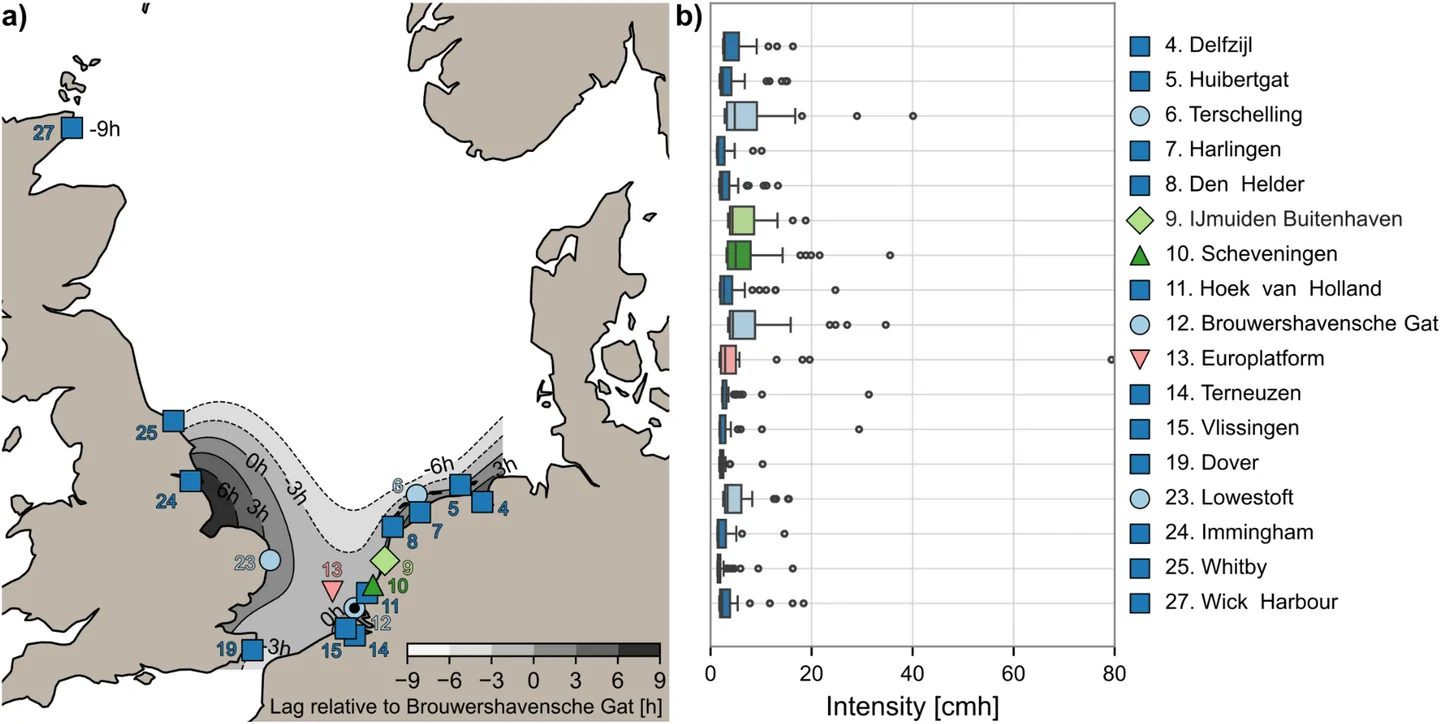
**a** Five spatial clusters and the interpolated lag of event maxima between stations, relative to Brouwershavensche Gat (station no. 12, marked with a black dot). **b** Distribution (box-plot) of event intensities at each station with a denoted spatial cluster symbol and station number

After removing data gaps and restricting the dataset to the common overlapping period among the 17 selected stations, a total of ~ 17 years of data per station remained for analysis. Events occurring within this period were grouped into 350 temporal groups, using the cross-correlation lag. The total count includes all instances where at least one station recorded an extreme, even if others remained silent. The stations were then partitioned into 5 clusters (see clustering scores in the Supplementary Information). The clusters and the corresponding distribution of cumulative intensity per station are illustrated in Fig. 5, excluding intensities equal to 0 cmh (indicating that the event did not occur at a station). The spatial clusters are described as follows:

1. *Light-green diamond*: This spatial cluster contains a single station, IJmuiden Buitenhaven, situated within a harbour. This station recorded high-intensity HF events (Fig. 5b).
2. *Green triangle*: Also comprising a single station, Scheveningen, that is similarly located within a harbour. This station recorded high-intensity events comparable to IJmuiden Buitenhaven (Fig. 5b).
3. *Light-blue circles*: This spatial cluster contains three stations: Terschelling, Brouwershavensche Gat, and Lowestoft. While positioned along open coastlines, Brouwershavensche Gat lies behind a sandbar, and Lowestoft is proximate to a harbour. These stations also exhibited high HF event intensities (Fig. 5b).
4. *Dark-blue squares*: This is the most populous cluster with eleven stations situated in various settings; it is dominated by locations primarily recording low-intensity events (Fig. 5b).
5. *Pink inverted triangle*: This spatial cluster also contains a single station, Europlatform, positioned far offshore. As expected, this station recorded low intensities, though higher than some stations within the dark-blue squares cluster (Fig. 5b).

**Fig. 6:**
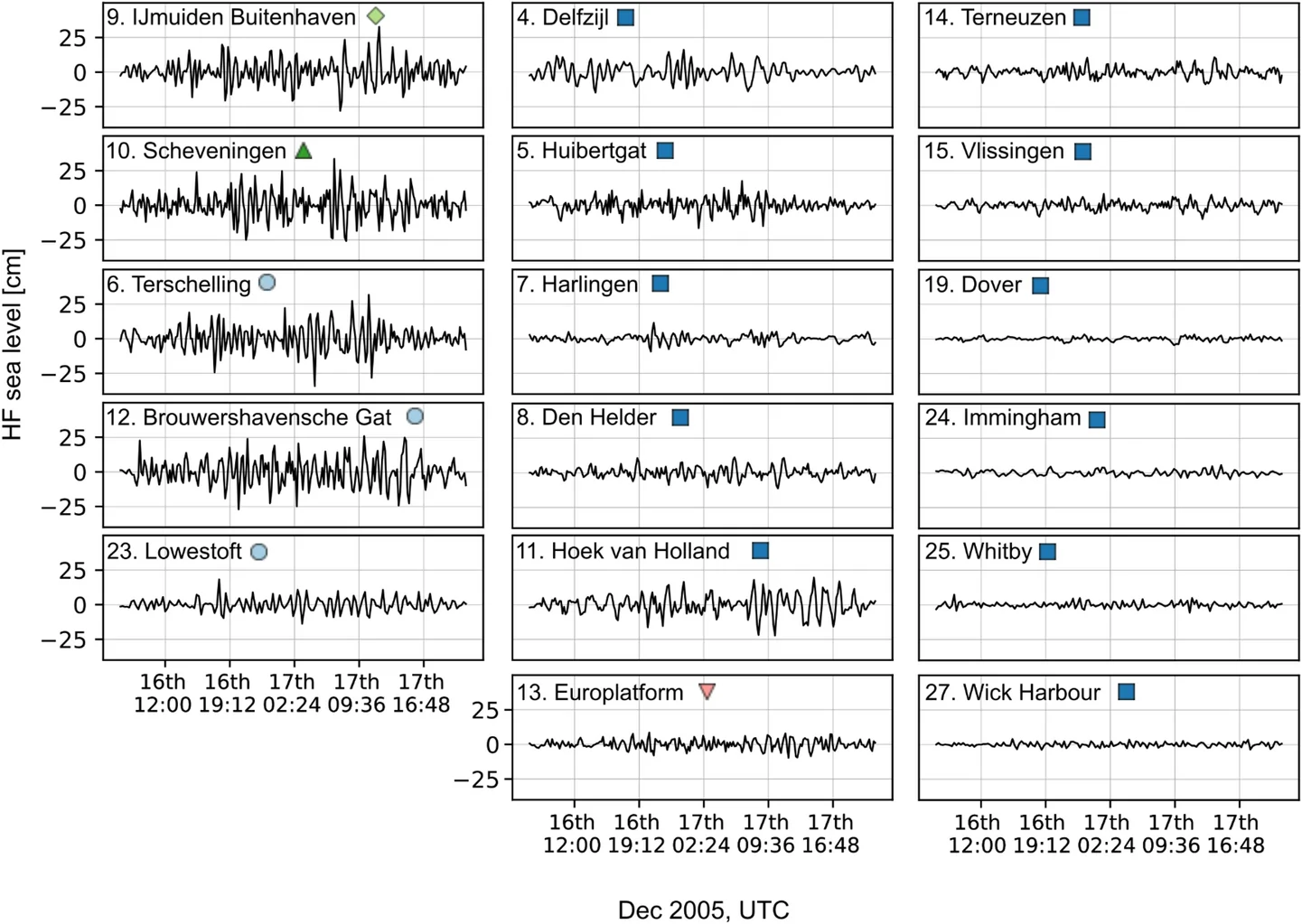
Hydrograph comparison for the December 2005 event. Stations are grouped according to the five defined spatial clusters (diamond, triangle, circles, squares, and inverted triangle) and station number, demonstrating distinct oscillation patterns observed in high-intensity versus low-intensity groups. Columns reflect an optimised layout for display purposes and carry no additional meaning

This clustering reveals that stations experiencing weaker HF event intensities were classified into a single cluster (dark-blue squares), whereas higher-intensity stations were distributed across three separate clusters (light-blue circles, green triangle, and light-green diamond). The only far-offshore station was separated from the other stations (Europlatform; pink inverted triangle). Since events at IJmuiden Buitenhaven and Scheveningen had intensities comparable to the events at stations marked with light-blue circles, and Europlatform had intensities similar to the intensities at stations marked with dark-blue squares, their classification into separate clusters was likely driven by temporal co-occurrence patterns and differences in HF event hydrographs (i.e., event evolution profiles). Figure 6 illustrates these hydrograph shape distinctions using one event; the first three clusters demonstrate higher oscillations. It is important to note that this example represents one of 350 temporal groups, and inter-event variability may yield differing degrees of similarity. The clustering reflects median behaviour across all temporal groups.

Moreover, the clustering reflects the pattern of 99.99th-percentile thresholds used to identify HF extreme events, as stations with comparable thresholds, and thus comparable intensity characteristics, clustered together. These distinctions underscore the dominant influence of local environmental settings in shaping the hydrograph, timing, and magnitude of HF extremes. However, stations in the dark-blue square cluster included a variety of environmental settings, including both open-coast and harbour locations. These results suggest that these stations do not exhibit the resonance effects required for the amplification of HF events. This is examined in more detail in the Discussion chapter.

Figure 5 also illustrates the time lag of HF maxima between stations, using Brouwershavensche Gat as the reference station. Negative values indicate that a given station reaches its maxima before the reference station. The findings indicate that the maxima were observed in the north of the UK and the Strait of Dover before their occurrence in the eastern UK (Lowestoft and Immingham) and the Wadden Sea (e.g., Delfzijl). Notably, the time lag distribution was similar regardless of the reference station selected. The estimated lag with Wick Harbour was approximately -9 h. However, due to the sparsity of observations in the open North Sea, the − 9 h isochrone was removed from Fig. 5, as it is likely an artefact of interpolation. Additional long-term records along the Norwegian coast would have been valuable to better constrain the propagation of HF maxima. However, the records from Maloy and Tregde were too short to be included in the spatial analysis. The limited availability of long-term data thus constrains the ability to provide a more detailed and accurate assessment of the lag behaviour of HF maxima along the North Sea coast; eleven stations were excluded from this analysis, resulting in a dataset largely comprising locations along the Dutch coast and, to a lesser extent, the UK coast.

### Event types

**Fig. 7:**
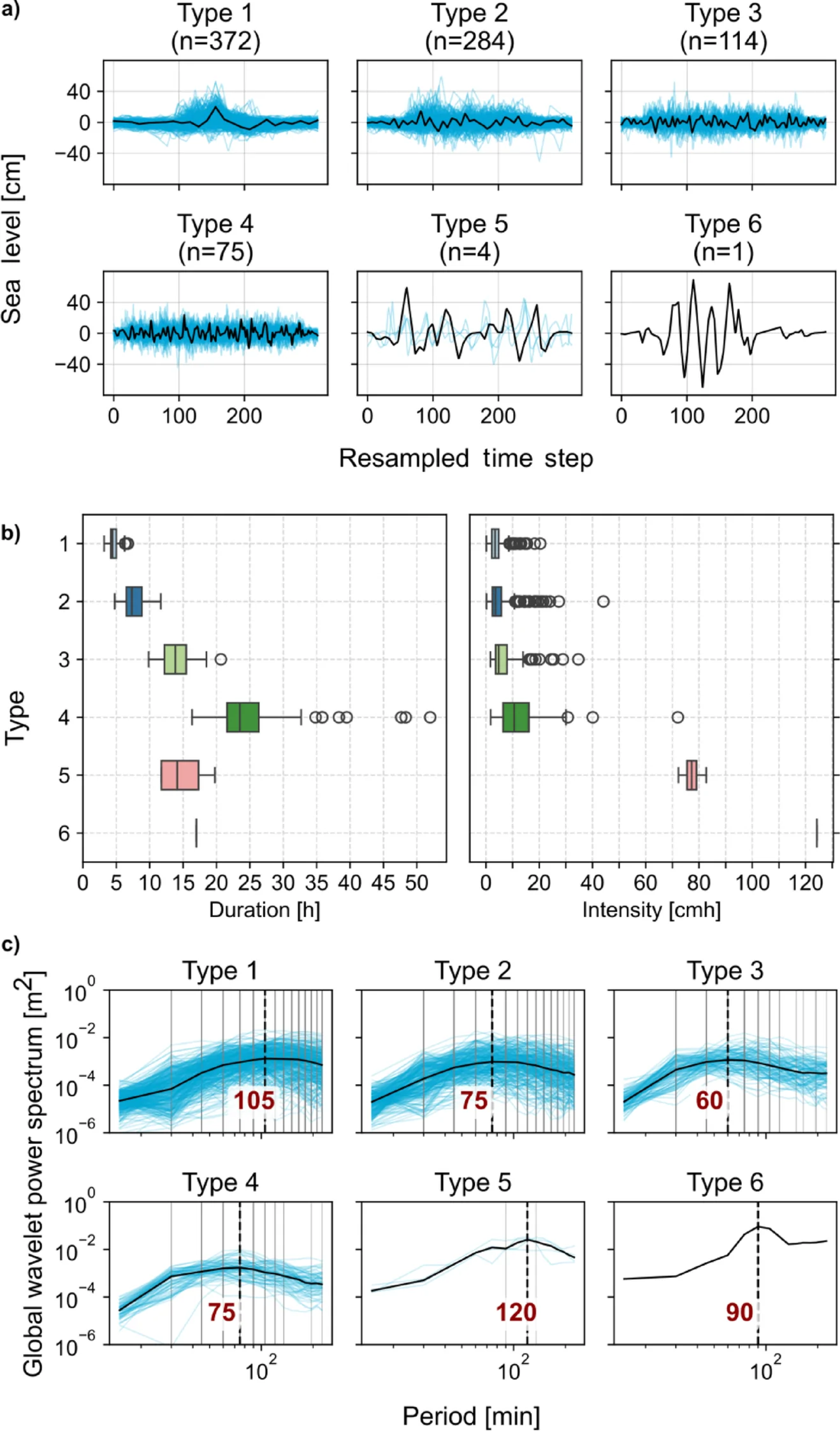
**a** The 6 identified event types, with the indicated number of events within each cluster. The cluster centroids are highlighted in bold black. The x-axis represents the resampled time step, not a specific time unit. The time series within a type do not align perfectly, as DTW clustering allowed for peak timing mismatch. **b** Distributions of event duration (*left*) and cumulative intensity (*right*) for each event type. **c)** The global wavelet power spectra for all time series within each event type. The median is shown in black. For each series, the dominant period is indicated by a dashed grey line, while the median dominant period is emphasised with a bold black dashed line and labelled with its value in minutes

In total, 850 events were classified under 6 types. The event types are described below, and the results are illustrated in Fig. 7.

Type 1: This type included 372 out of 850 events (44%), making it the most frequent type. Type 1 events were characterised by short durations (less than 10 h) and low intensities (Fig. 7b). Their time series exhibited small-amplitude oscillations with a relatively simple shape, often marked by a triangular peak (Fig. 7a). Analysis of the global wavelet power spectra revealed variability within the dominant period, with a median value of 105 min (Fig. 7c).
Type 2: A total of 284 events (33%) fell into Type 2. These events displayed slightly larger variance compared to Type 1, while still being predominantly short in duration and low in intensity (Fig. 7b). In contrast to Type 1, Type 2 events tended to last longer and often exhibited multi-modal oscillatory behaviour (Fig. 7a). The median dominant period was 75 min (Fig. 7c).
Type 3: Type 3 comprised 114 events (13%). These events were generally longer lasting, with durations between 10 to 20 h (Fig. 7b). Despite their increased duration, the intensities were comparable to those of Type 2, as Type 2 events occasionally reached higher peak amplitudes (Fig. 7a). The median dominant period for Type 3 was 60 min, indicating a shift towards higher-frequency oscillations (Fig. 7c).
Type 4: This type consisted of 75 events (9%), and it was characterised by the longest durations and higher intensities among the more frequently occurring types (Fig. 7b). The median dominant period was 75 min (Fig. 7c), the same as observed for Type 2, suggesting similar oscillations but with greater persistence.
Type 5: Only 4 events were classified as Type 5, representing rare but high-impact oscillations. These events had moderate durations, lasting on average 14 h, but exhibited very high intensities, exceeding 80 cmh (Fig. 7b). The median period of this event type was 120 min (Fig. 7c). These four events were recorded at Sheerness (21 Nov 2016 and 21 Dec 2007), Wick Harbour (7 Feb 2015) and Leith (14 Feb 2020).
Type 6: Type 6 represented a single outlier event and corresponded to the most extreme oscillation observed in the dataset. This event was recorded at Sheerness on 12 Nov 2009, lasted 17 h, and reached a maximum amplitude of 70 cm, resulting in an intensity exceeding 120 cmh (Fig. 7a, b). The median dominant period was 90 min, and the time series showed exceptionally large and coherent oscillations (Fig. 7a, c).

Overall, the classification revealed a clear progression from frequently occurring, weak oscillations (Types 1–2) to rare, high-intensity events (Types 5–6). While duration increased gradually from Type 1 to Type 4, the most extreme events were primarily distinguished by their intensity and spectral coherence rather than by duration alone. Notably, Types 2 and 4 shared a median dominant period of 75 min, suggesting similar underlying processes. However, their different durations and intensities justified their separation into distinct event types, with Type 4 representing a more sustained form. The well-documented Dutch meteotsunami of May 2017 (Sibley et al. 2021) was classified as Type 1 across all stations that detected it (Brouwershavensche Gat, Scheveningen, IJmuiden Buitenhaven, Terneuzen, Den Helder, Hoek van Holland, and Huibertgat). The event at Sheerness on 12 Nov 2009 was not documented as a HF event. However, it coincided with a series of storms affecting the UK that month, bringing heavy rainfall and strong winds, which led to flooding in various areas (Met Office 2009).

**Fig. 8:**
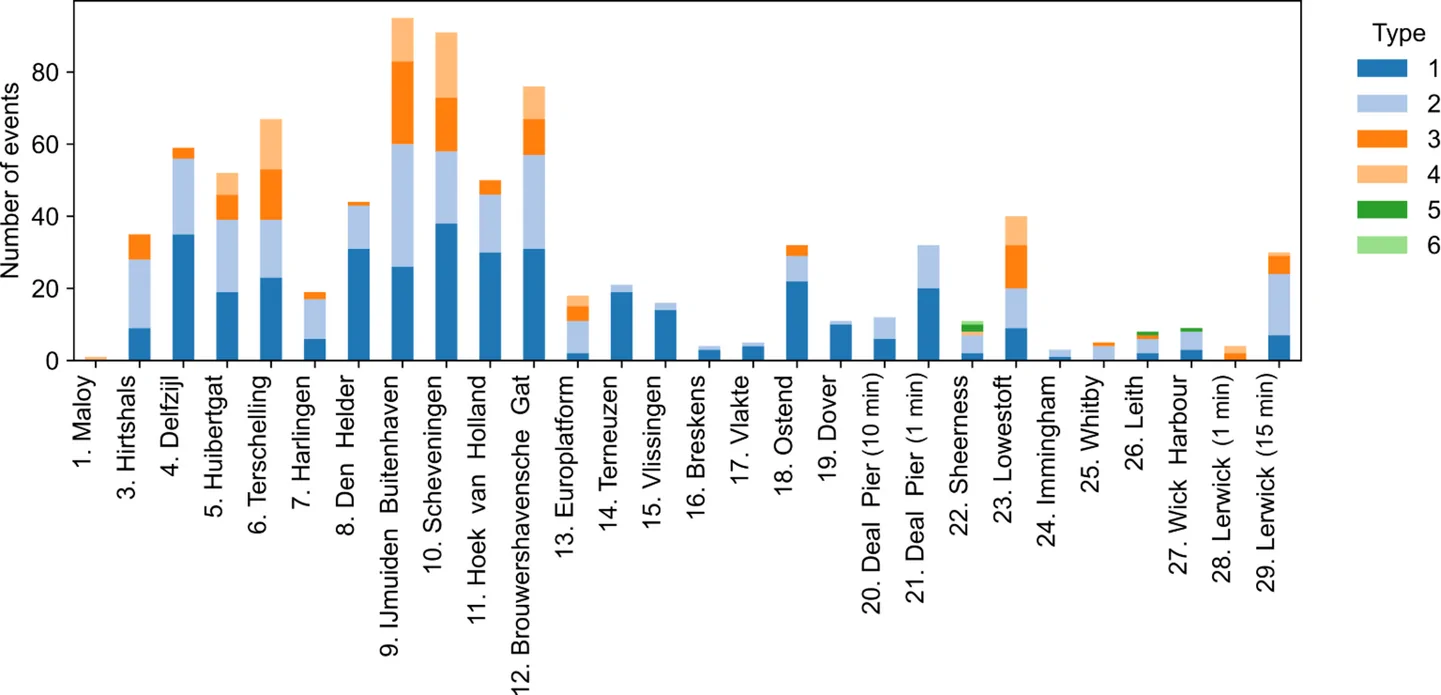
Distribution of events per station across event types

The MDA algorithm did not differentiate between stations and, in selecting the 850 most representative extreme events, excluded all events from the Tregde tide gauge. As noted earlier, this station exhibited a very low 99.99th-percentile threshold of only 2 cm. Figure 8 shows the total number of MDA-selected events per station and per cluster. Across all sites, the event record was dominated by Types 1 and 2, reflecting the prevalence of weaker, short-lived oscillations. Stations such as IJmuiden Buitenhaven, Scheveningen, and Brouwershavensche Gat recorded a higher total number of events. These were the stations that also had the highest 99.99th-percentile thresholds. However, they did not capture the high-intensity event types, Types 5–6. These rare, high-intensity events were spatially clustered at a limited number of locations, notably Sheerness, Leith, and Wick Harbour, where higher-type events occurred despite the comparatively modest total number of events. Additionally, several stations recorded fewer events that were almost exclusively low-intensity types, indicating that local conditions are generally not conducive to the development of strong HF events. This variation is likely related to measurement locations within harbour or bay geometries versus open coast settings, as further discussed in the Discussion chapter.

### Compound events

**Fig. 9:**
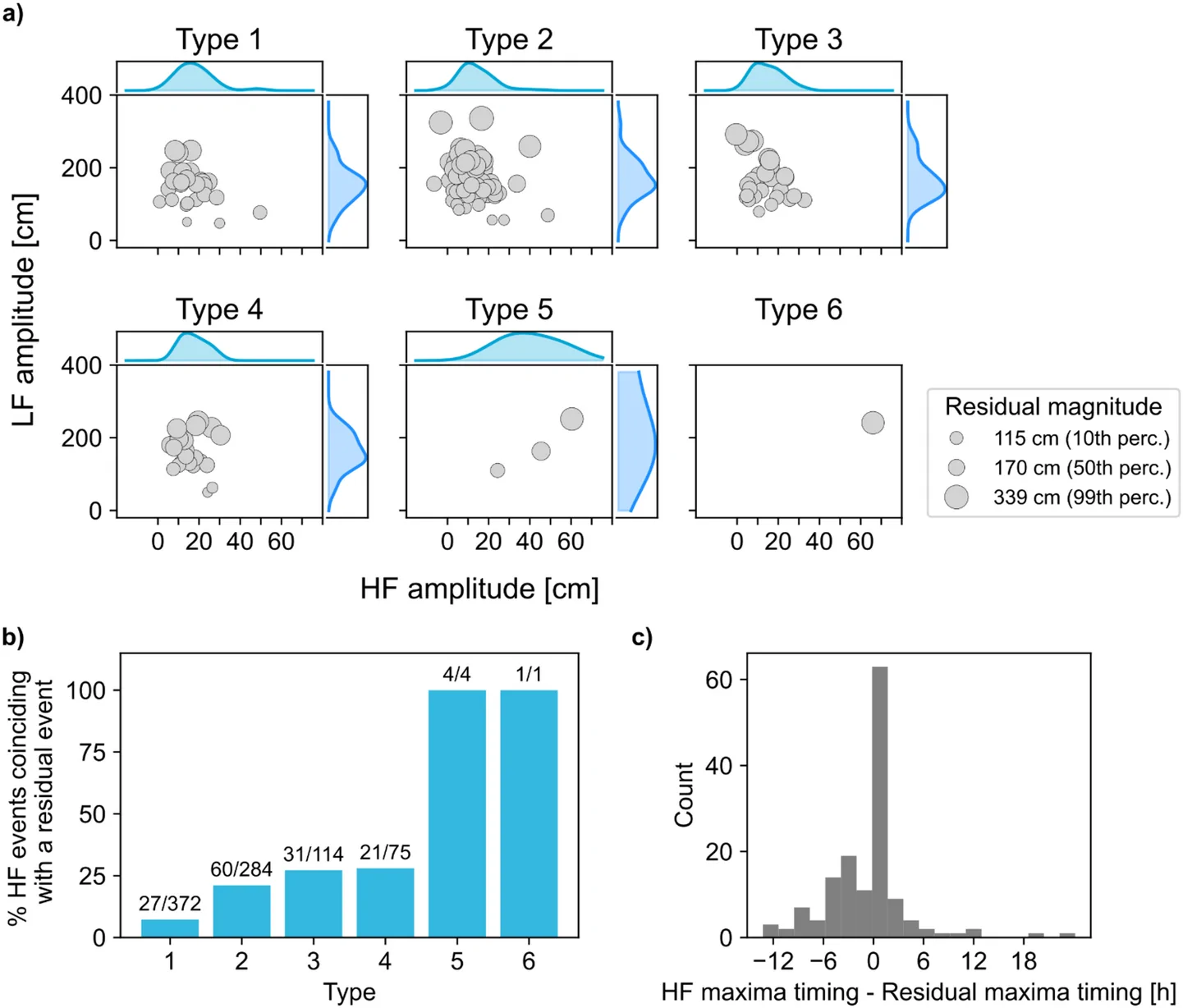
**a** Scatter plots of high-frequency (HF) versus low-frequency (LF) amplitudes for compound events of each event type. Marker size is proportional to residual magnitude, whose percentile values are computed over the entire residual series, not per station. Marginal density distributions of HF and LF amplitudes are shown along the top (HF amplitude) and right (LF amplitude) of each scatter plot. **b** Percentage of HF extremes coinciding with a residual extreme event per cluster, with counts shown above each bar. **c** Phase difference between the HF maxima and residual maxima for compound events across all clusters. A negative value indicates that the HF maximum occurred before the residual maximum

On average, 12.2% of the total 1,550 HF extremes at a given station coincided with residual extremes over the duration of an HF extreme. This proportion ranged from a maximum of 100% at Lerwick (1 min), to 23.8% at Harlingen, and 0% at Breskens (all values are listed in Table 2). Furthermore, the mean proportion of residual maxima amplitude attributable to the HF component during each compound event varied from 59% at Lerwick (1 min) to 1.9% at Tregde (not counting 0% stations; all values are listed in Table 2). However, the HF maxima did not always coincide with the residual maxima, as the HF signal can lead or lag the residual signal (Fig. 9c). In such cases, the HF maxima may present as a (smaller) secondary peak either before or after the main storm surge peak in the residual hydrograph (seen before in Fig. 3).

**Table 2:** The percentage of HF extremes coinciding with residual extremes and the mean HF component contribution (percentage) to the total residual amplitude per station

Station	% HF extremes coinciding with residual extremes	Mean HF contribution (% of residual amplitude)
*Maloy*	19.4	5.8
*Tregde*	7.3	1.9
*Hirtshals*	10	11.6
*Delfzijl*	13	5.3
*Huibertgat*	10.3	5.7
*Terschelling*	10.3	7.7
*Harlingen*	23.8	3.9
*Den Helder*	2.7	7.6
*IJmuiden Buitenhaven*	11.2	11.2
*Scheveningen*	18.3	10.3
*Hoek van Holland*	10.8	9.6
*Brouwershavensche Gat*	19.5	10.8
*Europlatform*	4.5	4.3
*Terneuzen*	3.6	9.4
*Vlissingen*	5.9	6.6
*Breskens*	/	/
*Vlakte*	5.9	8.6
*Ostend*	/	/
*Dover*	5.8	9.9
*Deal Pier (10 min)*	/	/
*Deal Pier (1 min)*	2.2	12.3
*Sheerness*	6.3	6.9
*Lowestoft*	11.1	6.3
*Immingham*	17.5	3.9
*Whitby*	13.8	5.2
*Leith*	7.7	13.4
*Wick Harbour*	11.8	29.3
*Lerwick (1 min)*	100	59
*Lerwick (15 min)*	/	/

At IJmuiden Buitenhaven (green diamond in Fig. 5), Scheveningen (dark-green triangle in Fig. 5), and Brouwershavensche Gat (light-blue circle in Fig. 5), notable for a higher representation in the MDA subset and the most intense HF events, the HF maxima contributed, on average, ~ 11% to the observed residual maxima. At Wick Harbour (weak-intensity cluster), the HF component accounted for an average of 29.3% of the residual maxima, in contrast to the remaining stations from the weak-intensity cluster (dark-blue squares in Fig. 5), which had an average contribution of 6.7 ± 2.3%. Stations in the light-blue circle cluster showed an intermediate HF contribution, with an average of 8.3 ± 2.3% (light-blue circles in Fig. 5). Due to insufficient data length (less than 10 years), Lerwick could not be included in the spatial clustering analysis; nonetheless, the Lerwick (1 min) exhibited a 99.99th-percentile level of ~ 21 cm, aligning it with the high-intensity event clusters. At this station, all HF extremes coincided with residual extremes, i.e., all HF extremes are also compound extremes.

#### Characteristics of compound events across event types

Compound events were further analysed per event type. Figure 9 illustrates the relationship between the HF, LF, and residual series across the 6 identified event types. The panels visualise the amplitude relationships, co-occurrence statistics, and timing offsets between the HF and residual extremes.

Across Types 1–4, HF amplitudes clustered around 10–20 cm and generally remained below 40–50 cm, whereas LF amplitudes reached higher values (100–300 cm). This pattern indicates that compound events in these types were primarily driven by LF oscillations, with HF extremes acting as secondary contributors. The coincidence rate between HF extremes and residual extremes was low for Type 1, with only 27 of 372 HF events (7%) being compound events (Fig. 9b). Across Types 2–4, this rate is moderate, with an average of ~ 25%. Together, these four types accounted for the majority of HF events, but only a limited fraction were compound events.

In contrast, Types 5 and 6, which contain only a few events, displayed distinct behaviour. Type 5 featured higher HF amplitudes (exceeding 60 cm), but paired with a wide range of LF amplitudes. Type 6 was marked by extreme HF amplitude coinciding with large LF amplitude and high residual magnitude. This type represents a rare but highly energetic compound event in which all components were simultaneously extreme. Notably, all HF events in Types 5 and 6 coincided with residual extremes (4/4 and 1/1 events, respectively; Fig. 9b), indicating a regime of strong process coupling.

Residual magnitudes further indicate that Types 2 and 3 contained the strongest compound events, largely dominated by the LF component. The ten highest HF contributions were associated with events at Lerwick (including both tide gauges), Wick Harbour, IJmuiden Buitenhaven, and Scheveningen. The largest absolute HF event, however, occurred in Type 6. This was a singular event at Sheerness, where the HF maximum reached 70 cm but did not coincide with the residual maximum. During this event, at the time of the residual maximum, the HF contribution was 66 cm, representing 21% of the residual value.

The temporal relationship between HF and residual maxima is illustrated in Fig. 9c. The distribution of phase differences between HF and residual maxima was generally centred close to zero hours, indicating that for most events the two components occurred with minimal time lag. Nonetheless, there were cases exhibiting both negative (HF leading) and positive (HF lagging) phase differences, with a greater tendency for the HF signal to lead. This variability suggests that HF extremes do not always occur precisely at the time of the residual maxima. Rather, HF maxima may precede or follow, giving rise to secondary peaks within storm surge hydrographs, as previously noted.

## Discussion

### Spatial differences

The results revealed consistent spatial differences in extreme HF events across stations, aligned with the established understanding of their driving mechanisms. It is known that extreme HF oscillations tend to occur at distinct coastal “hot spots”, where local bathymetric and topographic configurations promote strong amplification and resonant responses (e.g., Rabinovich 2009). Mesoscale weather systems, such as cold fronts and convective cells, have been previously recognised as triggers of meteotsunamis and seiches in the North Sea (Williams et al. 2021; de Jong et al. 2003, 2021; Sibley et al. 2021). Their propagation speed and pathway determine whether, and to what extent, the HF signal is amplified along the shelf (e.g., Sibley et al. 2021), with further amplification related to site-specific coastal geometry and resonance characteristics. Thus, tide gauge positioning largely determines how and to what extent local characteristics will be included in the observed signals.

Mean HF extreme amplitudes across the study area averaged 15.7 ± 7.6 cm, ranging from 2.5 cm at Tregde (archipelago setting, 10-min sampling) to 33 cm at Lerwick (channel setting, 1-min sampling) (Fig. 11). Stations located within long harbours or channels show amplified events due to wave trapping and resonance effects (e.g., Rabinovich 2009), as demonstrated by, e.g., IJmuiden Buitenhaven in this study. Bays similarly respond to the incoming energy (e.g., Rabinovich 2009), as observed, e.g., at Wick Harbour. In contrast, exposed locations generally capture the full incident wave field with minimal local influence (e.g., Ruić et al. 2023), here exemplified by Europlatform. The three largest recorded events occurred at Sheerness (Type 6, 70 cm), IJmuiden Buitenhaven (Type 2, 61 cm), and again at Sheerness (Type 5, 60 cm). Beyond these individual records, strong HF extreme events in the dataset clustered along the Dutch coast, particularly at Scheveningen, Brouwershavensche Gat, and IJmuiden Buitenhaven. This pattern likely reflects both coastal geometry and the nearby shallow continental shelf with its gentle slope, factors known to amplify HF oscillations. These findings are consistent with de Jong and Battjes (2004).

This difference in station behaviour across local environmental settings (e.g., harbour versus open coast) was also confirmed by the identified spatial clusters. In addition, these clusters captured variations in event-type distributions as well. Across all sites, weaker, short-lived oscillations prevailed, as reflected by the dominance of Types 1 and 2. Stations forming the higher-intensity clusters (light-blue circles: Terschelling, Brouwershavensche Gat, and Lowestoft; green triangle: Scheveningen; light-green diamond: IJmuiden Buitenhaven) showed the highest numbers of Type 3 and 4 events. Types 5 and 6 represent a more localised pattern, and were recorded only at Sheerness, Leith, and Wick Harbour. Among these, only Wick Harbour was included in the spatial clustering analysis. There, it grouped with the weak-intensity stations (dark-blue squares) because, like the other stations in that cluster, it was dominated by Type 1 and 2 events despite hosting some high-intensity cases. This variation is again related to tide gauge positioning: IJmuiden Buitenhaven, Scheveningen, and Wick Harbour are situated within harbour environments; Sheerness lies in an estuary; and Leith is positioned in a bay.

However, the relationship between local geometry and event intensity is not straightforward. Three open coast stations (Terschelling, Brouwershavensche Gat, and Lowestoft) emerged as a distinct spatial cluster (light-blue circles) characterised by amplified signals and a high incidence of Type 3 and 4 events. This showed that, in the North Sea, signals can also be amplified at open coast stations. Accordingly, the port of Terneuzen recorded a lower threshold (14 cm) than the more exposed station Terschelling (17 cm). This suggests that while sheltered locations can amplify signals through resonance, they can also suppress them if the local geometry does not support the resonant modes of the incoming waves. Additionally, open-coast stations may benefit from broader shelf-scale effects, such as shoaling and edge waves.

**Fig. 10:**
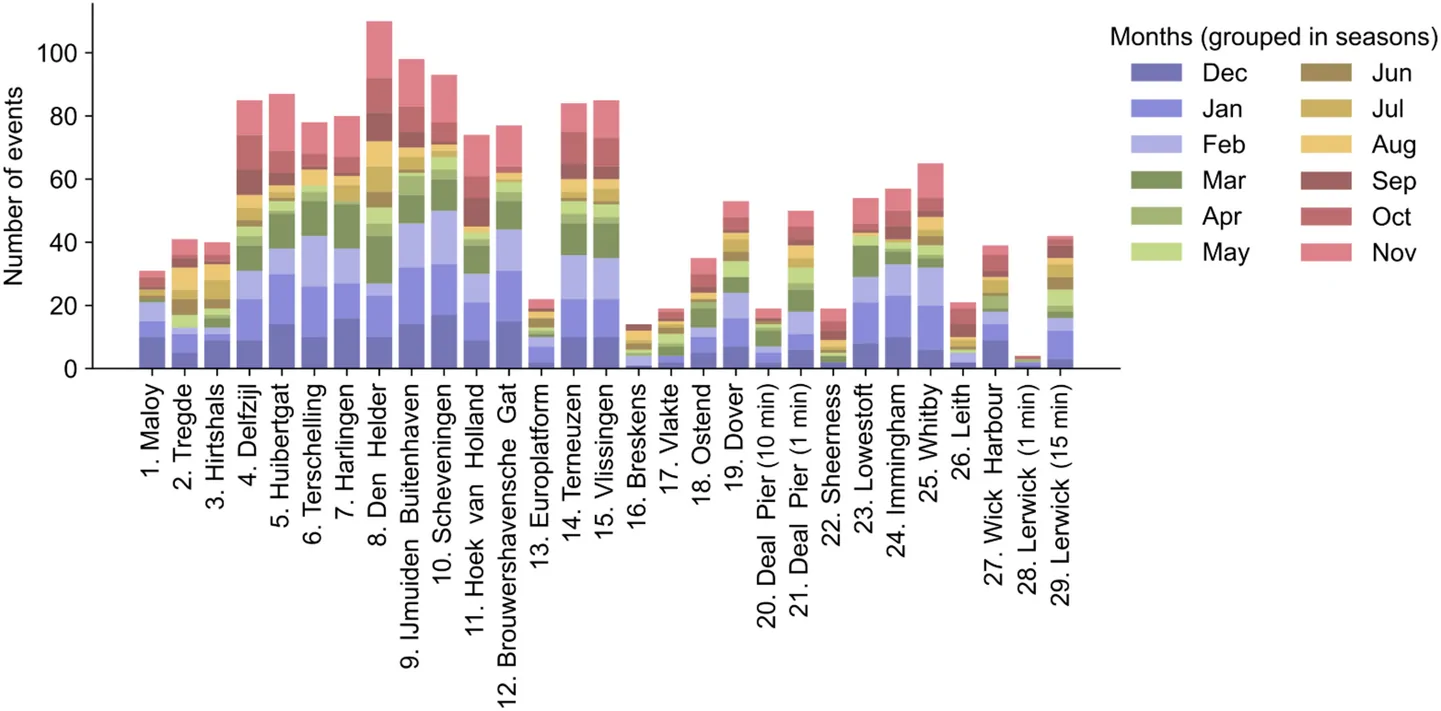
Distribution of HF extremes per month per station. The months are colour-coded per season: blues for winter (DJF); greens for spring (MAM); yellows for summer (JJA); and reds for autumn (SON)

The timing and characteristics of atmospheric drivers help explain the observed seasonality of HF extremes. Seasonal analysis (Fig. 10), averaged across stations, showed a winter peak (41.4 ± 12.45%) in the number of events, followed by autumn (26.5 ± 9.5%), spring (18.4 ± 7.5%), and summer (13.8 ± 10.8%). This reflects the importance of cold-season weather systems for generating HF extremes. In the cold season, the cold polar air over relatively warm seawater generates unstable atmospheric layers and supports the generation of mesoscale convective cells capable of generating HF extremes (de Jong and Battjes 2004; Williams et al. 2021). In contrast, summer months experience fewer HF extremes, likely due to fewer convective events. This seasonal behaviour is consistent with de Jong et al. (2021), who identified winter meteotsunami events as the primary concern for coastal defence assessments across the Dutch coast.

However, the seasonal distribution of HF extremes varied across stations, as some (e.g., Breskens and Tregde) recorded more events during the warmer than during the colder months. Events in warmer months may pose greater risks as more people engage in near-coastal daily and recreational activities. An interpretation of this finding would require analysis of atmospheric data, as no clear pattern emerged; however, this is beyond the scope of the present study and should be addressed in future research.

The analysis of HF extremes lag indicated that the HF maxima are normally observed first in the north of the UK, then along the Strait of Dover, and subsequently in the eastern UK and eastern North Sea. This pattern suggests that the observed events are influenced by atmospheric processes propagating from the west towards the east. Throughout the North Sea basin, the wind-driven residual circulation is mainly counterclockwise, reflecting the influence of prevailing westerly and south-westerly winds. These wind patterns define the mean state of the surface atmospheric flow over the North Sea, which is partially governed by the pressure gradient between the Icelandic Low and the Azores High (Quante and Colijn 2016; Rubinetti et al. 2023). Additionally, Williams et al. (2021) found that the dominant synoptic weather feature at the time of meteotsunami detection in northwest Europe was an extratropical cyclone located north or west of the UK. They also showed that the mean lower- and mid-tropospheric wind conditions were favourable for the eastward propagation of mesoscale precipitation systems. The lag of HF event maxima observed in this study seems consistent with these mechanisms.

In addition, the dynamics of the North Sea are strongly influenced by astronomical tides. Tidal elevations propagate from the Atlantic and circulate counterclockwise as a Kelvin wave throughout the basin (Quante and Colijn 2016; Otto et al. 1990). Therefore, it is important to note that some residual tidal signal may still be present in the filtered series and thus contribute to the observed lag structure. Even after tidal removal, nonlinear tide–surge interactions can lead to incomplete separation of tidal and non-tidal components, particularly in shallow, macrotidal environments such as those found in the North Sea. Choi et al. (2014) demonstrated such nonlinear coupling, reporting tide-related amplitude changes of about 17% for meteotsunamis in the Yellow Sea. For the North Sea, Williams et al. (2021) showed that high-frequency wavelet features can persist at the M2 period and be modulated by the spring–neap cycle even after harmonic tidal removal and high-pass filtering. Consequently, part of the observed lag in HF extreme maxima may reflect phase-modulated tidal contributions that propagate counterclockwise through the basin. A detailed analysis of the interaction between HF extremes and the tidal signal was beyond the scope of this study; however, it would be a valuable direction for future research.

### Compound events

**Fig. 11:**
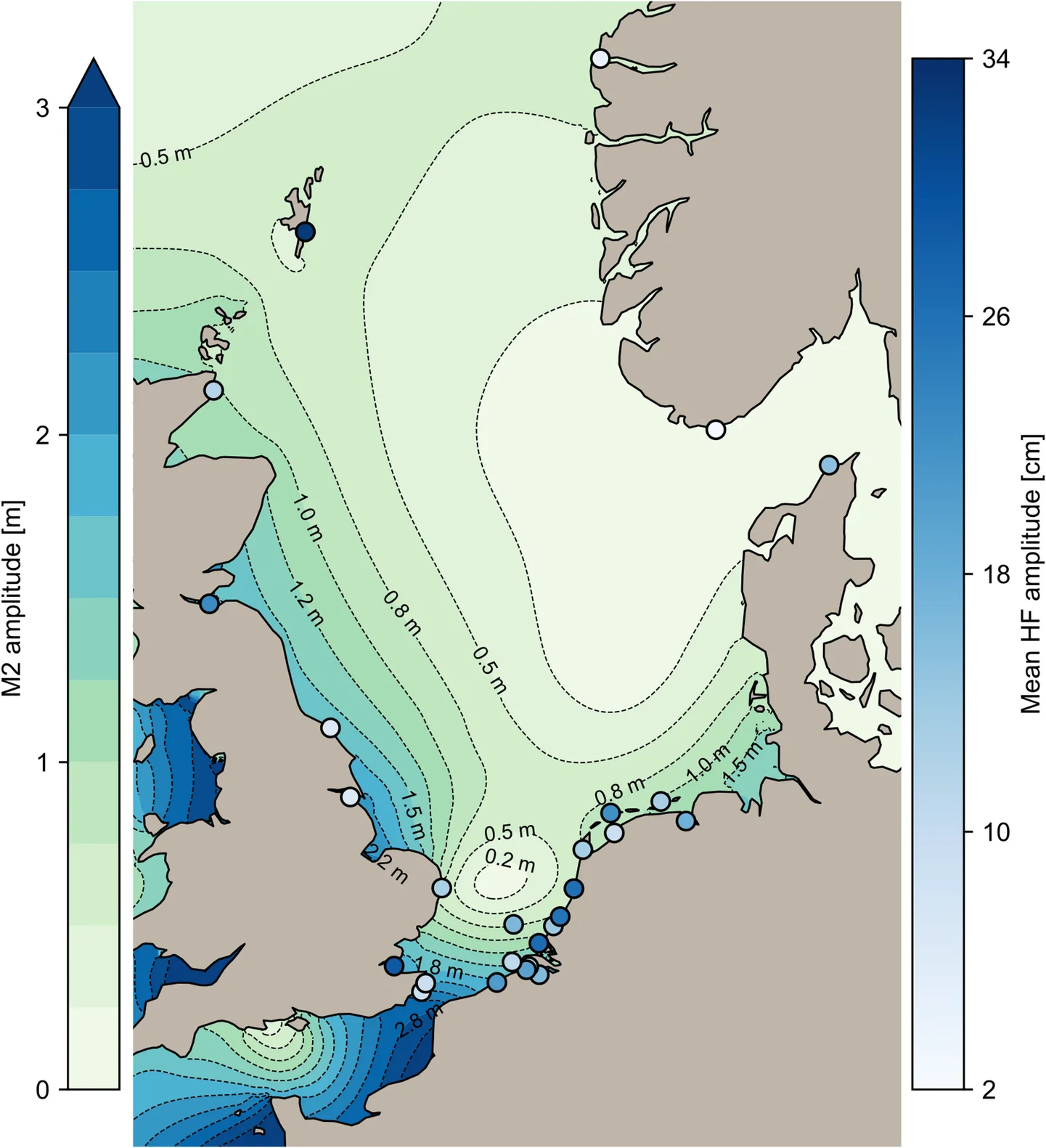
The North Sea tidal system for the M2 tide showing its amplitude plotted from the FES2022 ocean tide dataset (Carrere, 2022; CNES 2024), as well as the mean HF extreme amplitude per station

Although the maximum HF extreme reached 70 cm, 95% of all events remained below 30 cm (ranging from 71% at Leith to 100% at Tregde). Despite local amplifications, HF extremes in the study area generally represent a limited hazard because regional tidal ranges (1–4 m) (Nieuwhof et al., 2018; Proudman and Doodson 1924) are typically an order of magnitude larger than the mean HF oscillation heights (see Fig. 11). However, despite lower sea-level heights, HF oscillations can still produce strong localised currents, making the extreme HF events dangerous (Linares et al. 2016).

Moreover, HF extremes may contribute to residual events at the main surge peak or as secondary peaks, as it was found that the HF maxima did not always coincide with the residual maxima (Fig. 3, Fig. 9c). In many cases, the HF extreme preceded the residual maxima, pointing to differences in the generating mechanisms of HF oscillations and storm surges. Intense small-scale atmospheric pressure disturbances, capable of generating HF extremes, are sometimes associated with temperature fronts of extratropical cyclones (e.g., Rabinovich et al. 2022; Heidarzadeh et al. 2025). These disturbances can propagate faster than the parent storms themselves, allowing the HF oscillations to arrive earlier than the storm surge maximum. As a result, the atmospheric pathways and timing favourable for HF event generation may not always align with those producing the largest residual sea levels, potentially limiting the occurrence of fully compound HF–surge events. Nevertheless, secondary peaks that are distinct from the main surge can cause unexpected fluctuations in water levels, putting additional stress on coastal defences and infrastructure. This could challenge emergency preparedness, impact real-time decision-making, and heighten the risk of infrastructure failure (Bakker et al. 2025).

The spatial variability of compound events observed across the study area further supports the importance of local and regional settings. Prevalent atmospheric forcing characteristics, such as disturbance speed and pathway, influence where and when HF oscillations co-occur with residual extremes. However, atmospheric forcing conditions were not analysed in this study, such an investigation was outside the scope of the present work.

Overall, the events characterised by aligned timing of HF and residual maxima highlight a dynamical linkage between low- and high-frequency sea-level processes during compound extremes. This synchrony amplifies total water levels and elevates flood potential. In contrast, the occasional asynchronous behaviour reveals the complexity of these interactions and points toward further research on the causal mechanisms supporting extreme sea-level events. Taken together, these findings reinforce the need to consider both the magnitude and timing of interacting sea-level components when assessing compound flooding risks in coastal regions.

### Limitations

There is a relatively dense and long-term sea-level observational network at the coasts of the North Sea, but not all available records were sufficient for comprehensive spatial clustering. As a result, 12 stations were excluded from the spatial analysis due to their short or fragmented records. Moreover, the dataset temporal resolution varied between 1 and 15 min, and time series were retained at their original resolution during the extraction of extreme values. This heterogeneity in sampling frequency introduces a source of uncertainty, as HF extremes derived from coarser (e.g., 15-min) records likely significantly underestimate the true peak magnitudes. Consequently, comparisons of HF extremes across sites should be interpreted with caution, as differences may partly reflect sampling limitations rather than underlying physical variability. For example, stations with shorter sampling intervals tended to have higher 99.99th percentile thresholds. This was clearly illustrated at Lerwick, where mean HF extreme amplitude at the 1-min tide gauge was 33 cm, compared with 14 cm at the 15-min gauge, highlighting how temporal resolution influences estimated extremes. This illustrates a limitation relevant to the North Sea and similar data settings: analyses of HF extreme sea levels are still relatively scarce, predominantly because long-duration, high-resolution datasets have only recently become available (e.g., Zemunik et al. 2021; Balić and Šepić's SHELDA dataset, in review, 2025). The clustering methodologies adopted in this study require adequate temporal coverage for reliable results, and their application was thus constrained. Nevertheless, the present study provides valuable information in classifying extreme HF sea-level events and their spatial variation in the North Sea. As the observational infrastructure continues to expand and more HF records become available, more thorough analyses will be achievable.

Moreover, the driving mechanisms were not analysed in this study, as such an investigation was outside the scope of the present work. This limited the ability to fully explain the observed temporal and spatial variability between events and locations. Future studies integrating atmospheric reanalysis and pressure-field observations would help clarify the processes controlling HF variability and the conditions under which HF oscillations and storm surges may produce compound coastal hazards.

## Conclusions

This study analysed high-frequency (HF; T < 2 h) sea-level extremes along the North Sea coast using long-term, high-resolution tide gauge observations, applying advanced time series and spatial clustering analyses. The main findings can be summarised as follows:

- HF sea-level extremes in the North Sea are generally low relative to residual extremes and to tidal ranges, but they might still affect total sea levels and pose additional dangers, especially in warmer months when more people engage in near-coastal activities.
- The results revealed consistent spatial differences, reflecting the influence of site-specific bathymetry and coastal geometry, as well as the likely variability of regional atmospheric forcings. This variability is captured by 5 spatial clusters: stations with weaker HF event intensities were grouped into a single cluster, while stations with higher intensities were separated into three distinct clusters, capturing heterogeneous high-energy responses along the coast. In addition, the far-offshore station, Europlatform, formed its own cluster. The most intense events were concentrated along the Dutch coast. Some of the observed differences may also be affected by heterogeneity in temporal sampling resolution, which can lead to an underestimation of peak HF extremes at stations with coarser records.
- Cluster analysis identified 6 distinct event types, revealing more frequent, lower-intensity oscillations, and rare, high-intensity events. Dominant periods ranged from 60 to 120 min, underscoring a range of underlying drivers. Types 2 and 4 share the dominant period, but their separation is justified by differences in persistence and strength, with Type 4 representing more sustained events.
- Seasonal patterns showed a pronounced winter peak in extreme HF event occurrence, associated with synoptic-scale atmospheric disturbances, thereby confirming earlier findings in the North Sea.
- Compound events, where extreme HF events coincided with residual extremes, were relatively rare (averaging 12.2% of HF extreme events across stations), but still elevated the total sea levels when residual and HF maxima aligned temporally.
- The timing of HF maximum relative to the residual maximum at a given location was often closely matched; however, asynchronous peaks also occurred, which can arise as secondary maxima during residual extreme events (i.e., storm surges).
- Spatial clustering was limited by the availability and consistency of high-resolution sea-level records. As the observational infrastructure continues to expand and more HF records become available, future studies will be better positioned to provide detailed, region-wide assessments of extreme HF event behaviour.

In conclusion, this research highlights that to assess extreme sea levels, it is essential to consider both the magnitude and timing of different frequency components, as well as the coastal features that influence them. By characterising extreme HF events and their interactions with lower-frequency processes, this study contributes to improved risk frameworks and coastal management strategies amid sea-level rise and climate change.

Future work should extend this analysis by investigating the underlying mechanisms driving the variability of identified event types, as well as the spatial distribution of HF extreme events across the North Sea. Such efforts are necessary to achieve a more comprehensive physical understanding of HF extremes.

## Supplementary Information

Below is the link to the electronic supplementary material.

Supplementary Material 1

## Data Availability

The pre-processed data is publicly available through the 4TU.ResearchData repository at https://doi.org/10.4121/0899eca1-f918-41f0-b2da-1da4cd56a873. All global datasets used in this study are also available online. For access, please refer to the corresponding references mentioned in this article.

## Code availability

The codes will be uploaded to a GitHub repository: https://github.com/pupicvuriljm/Mixed-Clustering
