# Supplementary material for: Investigation of North Sea high-frequency sea-level extremes using long-term sea-level measurements
Source: Nat Hazards (Dordr). 2026 Aug 26;122(18):616. doi: 10.1007/s11069-026-08343-y (PMC13506592; doi:10.1007/s11069-026-08343-y)
Supplement: Supplementary file 1 [file 11069_2026_8343_MOESM1_ESM.pdf]

Investigation of North Sea high-frequency sea-level extremes using long-term sea-level measurements

Mia Pupić Vurilj[1*], Gozde Guney Dogan[1], Marijana Balić[2,3], José A. A. Antolínez[1], Jadranka Šepić[2],

[1]Delft University of Technology, Department of Hydraulic Engineering, Delft, Netherlands
[2]University of Split, Faculty of Science, Split, Croatia
[3]University of Split, Split, Croatia

*Corresponding author. E-mail: m.pupicvurilj@tudelft.nl (https://orcid.org/0009-0003-8719-3182);
Contributing authors: g.g.doganbingoel@tudelft.nl, (https://orcid.org/0000-0002-8638-8792); mbalic@pmfst.hr (https://orcid.org/0000-0002-1183-3214); j.a.a.antolinez@tudelft.nl (https://orcid.org/0000-0002-0694-4817); jsepic@pmfst.hr, (https://orcid.org/0000-0002-5624-1351)

Supplementary Information (SI)

Cross-correlation lag

(I) **Gap-aware segmentation**: Time series from the selected stations were divided into continuous, gap-free segments.
(II) **Cross-correlation analysis**: For each pair of stations, the segments were compared using cross-correlation to estimate the relative lag and correlation strength.
(III) **Statistical significance testing**: A permutation test was applied to each segment pair to compute p-values, assessing whether the observed maximum cross-correlation was statistically significant.
(IV) **Weighted lags**: Statistically significant lags ($p \leq 0.05$) were combined using a weighted median, where weights were proportional to both segment length and correlation magnitude.
(V) **Lag matrix**: The resulting median weighted lags were assembled into a full station-by-station lag matrix, summarising timing relationships across the stations.

Resampling in time

Distance-based clustering methods require all data points to have the same number of features. When clustering time series, this means that all series must have an identical number of time steps, because each time step is treated as a separate feature in the feature space. Since the events in the dataset have varying time steps (Δt) and durations (D), it was necessary to resample the time series to allow for comparison of the different event shapes.

Each event series was resampled to an equal number of time steps using the length of an event with the maximum value of $\Delta t \cdot D$ as the reference. This corresponded to 239 time steps for spatial clustering and 313 time steps for event-type clustering. The two clustering procedures had different numbers of time steps because they used different reference events. The reference events differed because the input data varied. For the spatial clustering, we used only the overlapping observation periods across the 17 selected stations and defined temporal groups.

Linear interpolation was applied to align data points with the new time steps. Since event durations differ, the resulting time step was event-specific. For example, in event-type clustering, an event lasting 10 hours had an interpolated time step of 10h/313 = 0.03 h = 1.8 min, while an event lasting 40 hours had a step of 40h/313 = 0.13 h = 7.8 min. As a result, long and short event shapes were directly compared during event-type clustering (**Fig. S1**). This is why duration was added as an equally weighted feature in event-type clustering. However, in spatial clustering, temporal groups were defined first, ensuring the comparison of equal-duration events. As a result, no compensation for the event duration was necessary. Below are two examples of events before and after resampling.

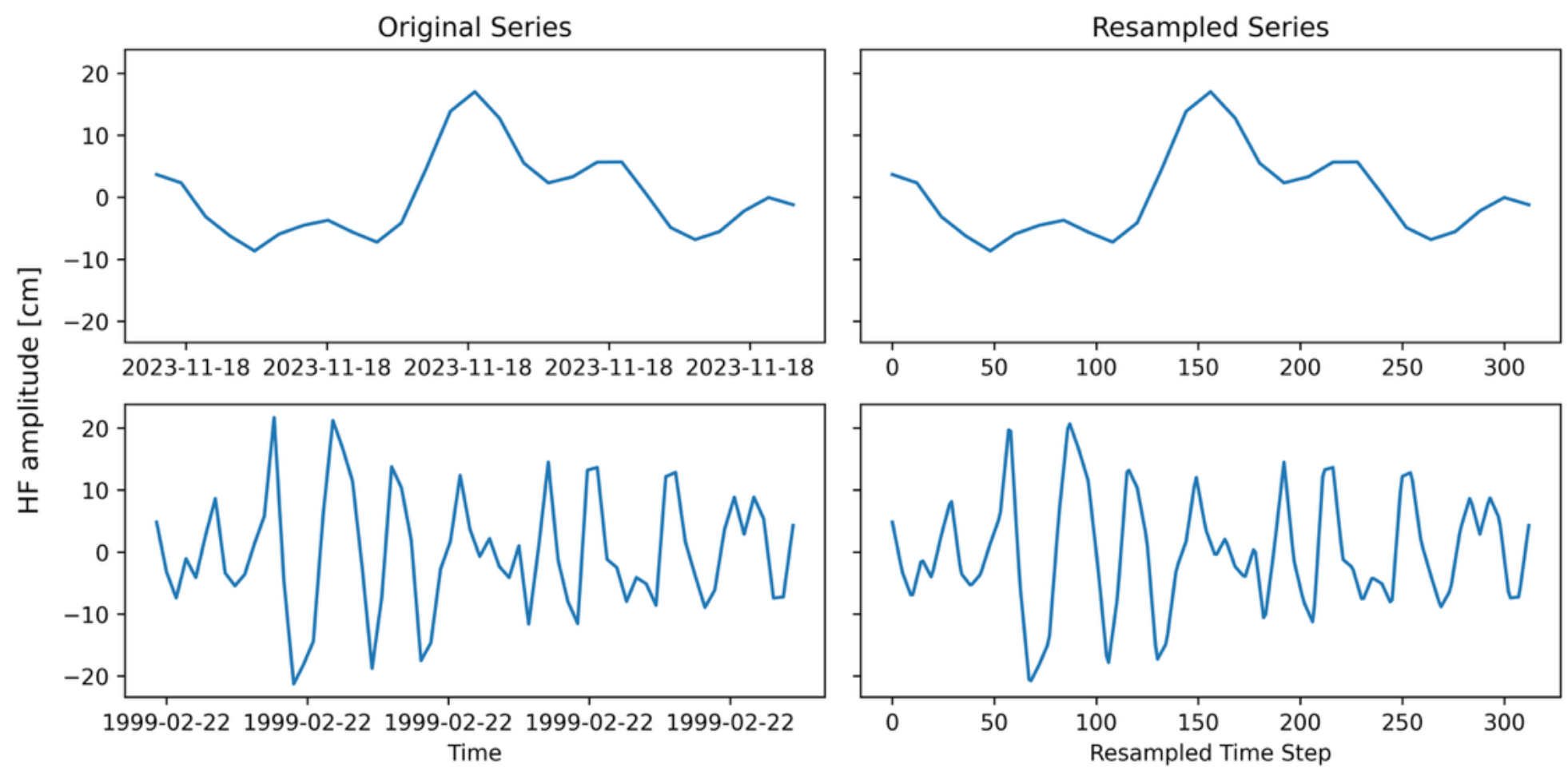

Fig. S1 Two events before and after resampling to 313 time steps for event-type clustering

Clustering features

Spatial clustering:

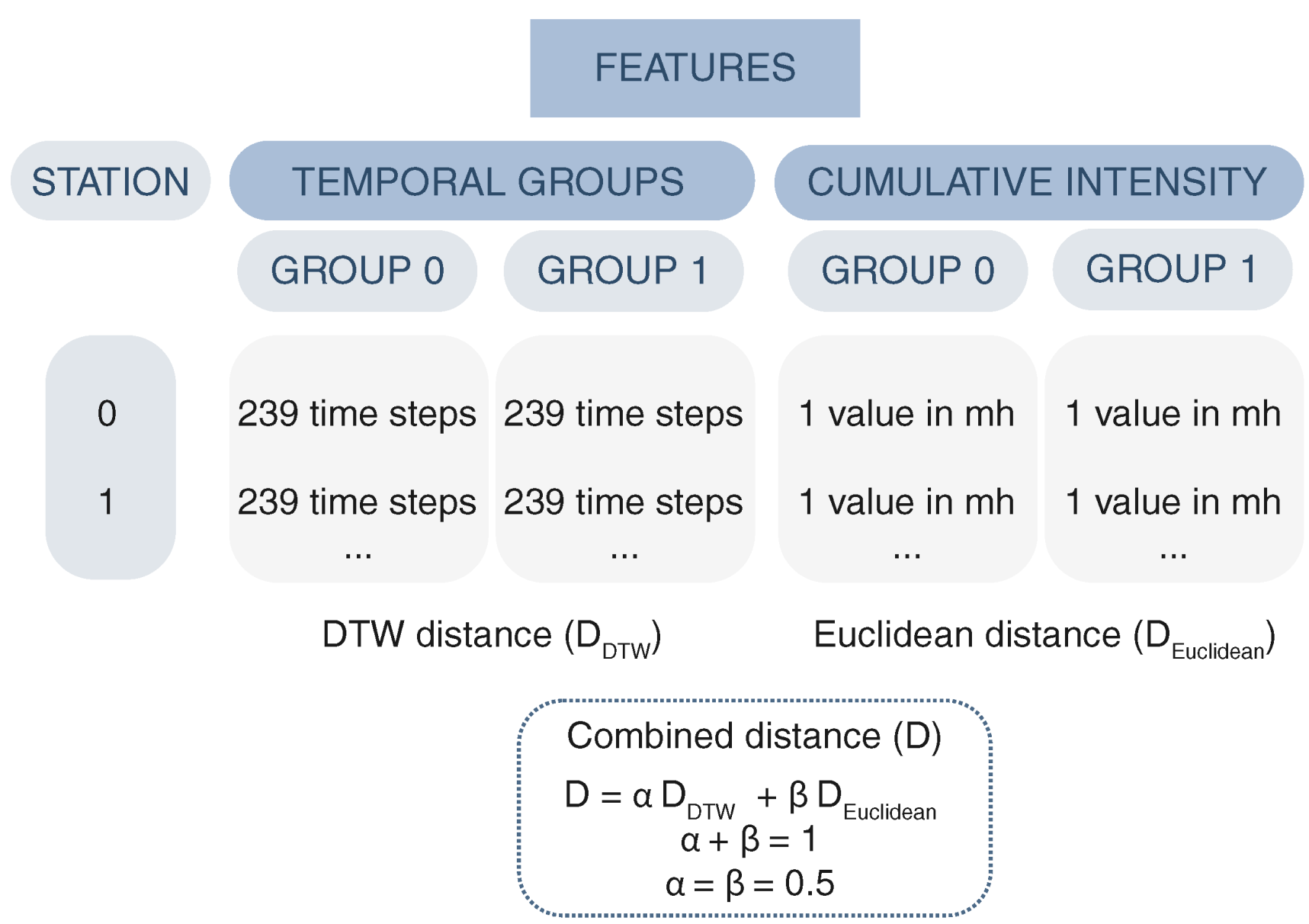

Event-type clustering:

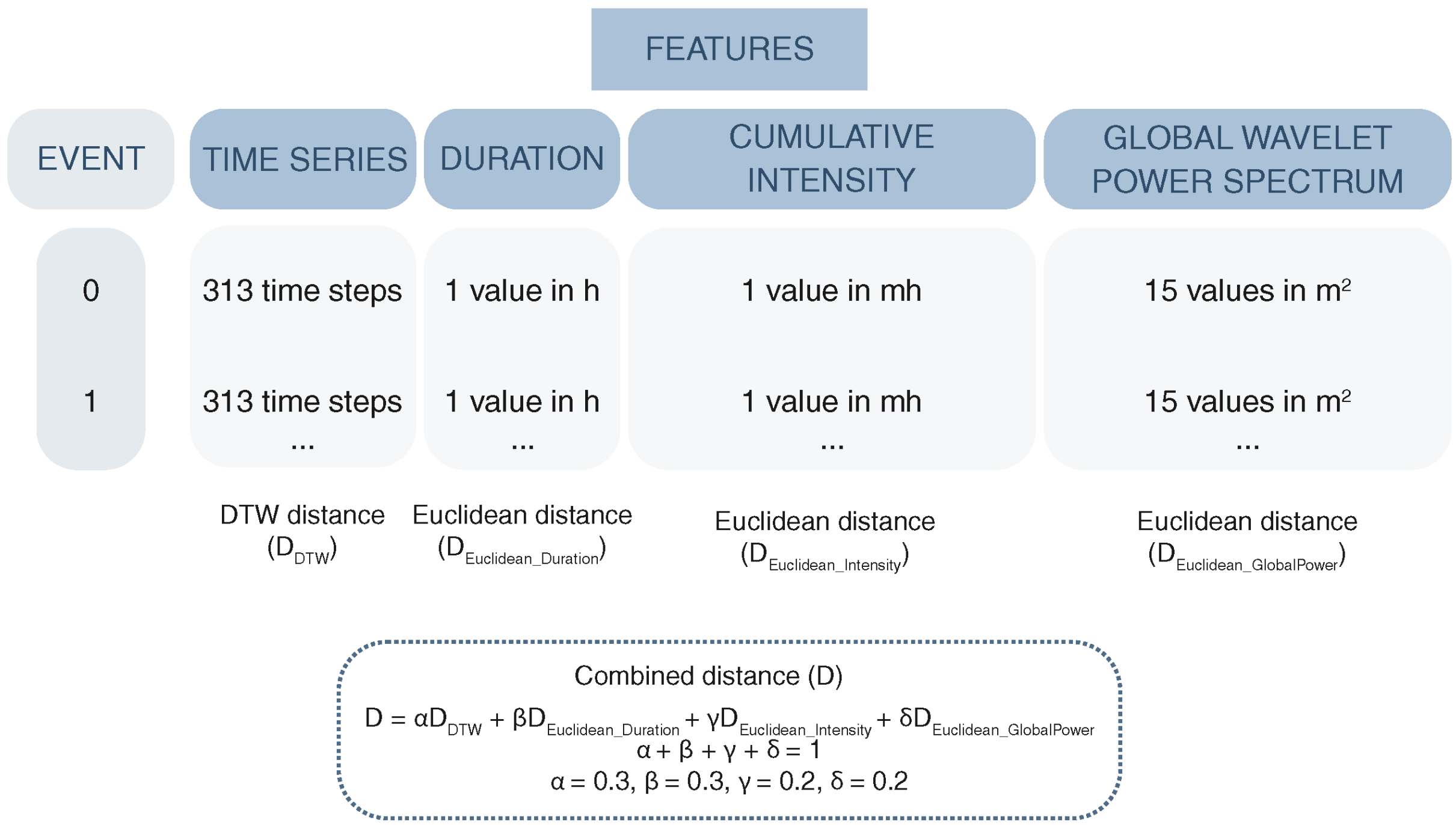

K-medoids

The K-medoids algorithm partitions a set of points $X = \{x_1, x_2, \dots, x_n\} \subseteq \mathbb{R}^d$ into K clusters $C = \{C_1, C_2, \dots C_K\}$ by minimising the distance of the points to the centres of the clusters (*cluster centroids*) (Kaufman et al., 1990). The centres form a set of medoids $M = \{m_1, m_2, \dots, m_K\}$, and the objective function to be minimised is defined as

$$Q(C, M) = \sum_{i=1}^{K} \sum_{x \in C_i} d(x, m_i) \quad (1)$$

where $d(x, m_i)$ denotes the distance between a point x and the medoid m_i of cluster C_i. K-Medoids works with any dissimilarity matrix, since it only relies on inter-observation distances and does not require a mean or median centre. Therefore, similar to K-means, K-medoids clusters data by finding central points, but instead of using the mean to calculate cluster centres, it uses the medoid. K-medoids is more robust to outliers, especially since the objective function is based on the sum of the actual distances instead of their squares.

To increase the performance of the clustering, the K-Medoids++ initialisation method was used. It is a specific way of choosing the initial centres for the K-medoids algorithm, based on K-means++ (Arthur and Vassilvitskii, 2007). The initial medoids are more separated than those generated by other methods, and speed and accuracy of convergence and clustering are increased.

Maximum Dissimilarity Algorithm (MDA)

The Maximum Dissimilarity Algorithm (MDA; Camus et al. 2011) is a method used to select a specified number of data points from a larger dataset in such a way that the selected points are maximally dissimilar to each other. Its objective is to construct a representative subset of size M from a database of size N. Given a data sample $X = \{x_1, x_2, \ldots, x_n\}$ of N n-dimensional vectors, the algorithm returns a subset $\{v_1, \ldots, v_m\}$ that reflects the overall diversity of the data.

The algorithm starts by initialising the subset with one vector, v_1, taken from the data sample. The remaining $M - 1$ vectors are then chosen iteratively. At each iteration, it computes the dissimilarity between each candidate point in the database and the current subset and transfers the most dissimilar point into the subset. This procedure repeats until M vectors have been selected. In this work, the *MaxMin* version of the algorithm has been considered. For example, if the subset is formed by R ($R \leq M$) vectors, the dissimilarity between the vector i of the data sample $(N - R)$ and the j vectors belonging to the R subset is calculated as

$$d_{i,j} = \|x_i - v_j\| \tag{2}$$

$$i = 1, \ldots, N - R$$
$$j = 1, \ldots, R$$

Subsequently, the dissimilarity $d_{i,subset}$ between the vector i and the subset R, is calculated as

$$d_{i,subset} = min\{\|x_i - v_j\|\} \tag{3}$$

$$i = 1, \ldots, N - R$$
$$j = 1, \ldots, R$$

Once the $N - R$ dissimilarities are calculated, the next selected data is the one with the largest value $d_{i,subset}$. This definition is taken from Camus et al. (2011).

In other words, the first selected vector $\{v_1\}$ is the point most dissimilar to the rest of the data and lies near the edge of the data space. Next, v_2 is chosen as the point most dissimilar to v_1, often lying near the opposite side of the data space. The algorithm then continues selecting v_3, v_4, and so on, drawing not only from the periphery but from the entire data distribution. As a result, the final subset is approximately uniformly distributed across the data space.

Clustering scores

Spatial clustering:

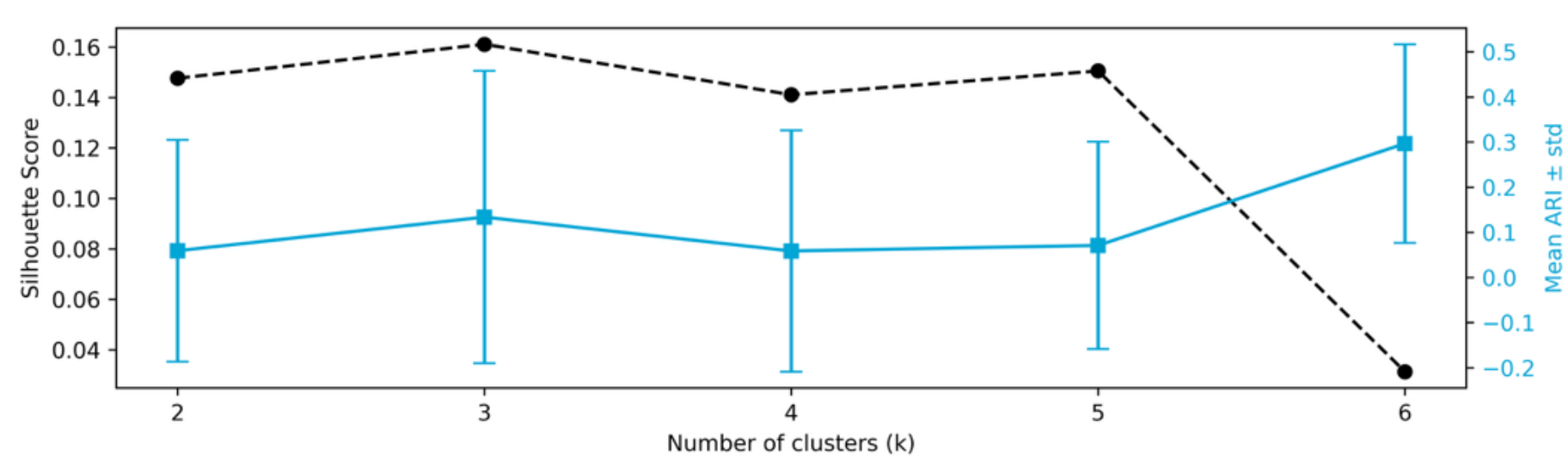

Chosen k = 5 according to the silhouette score and cluster stability (the Adjusted Rand Index, ARI), and after the analysis of the distributions of features used in clustering.

Event-type clustering:

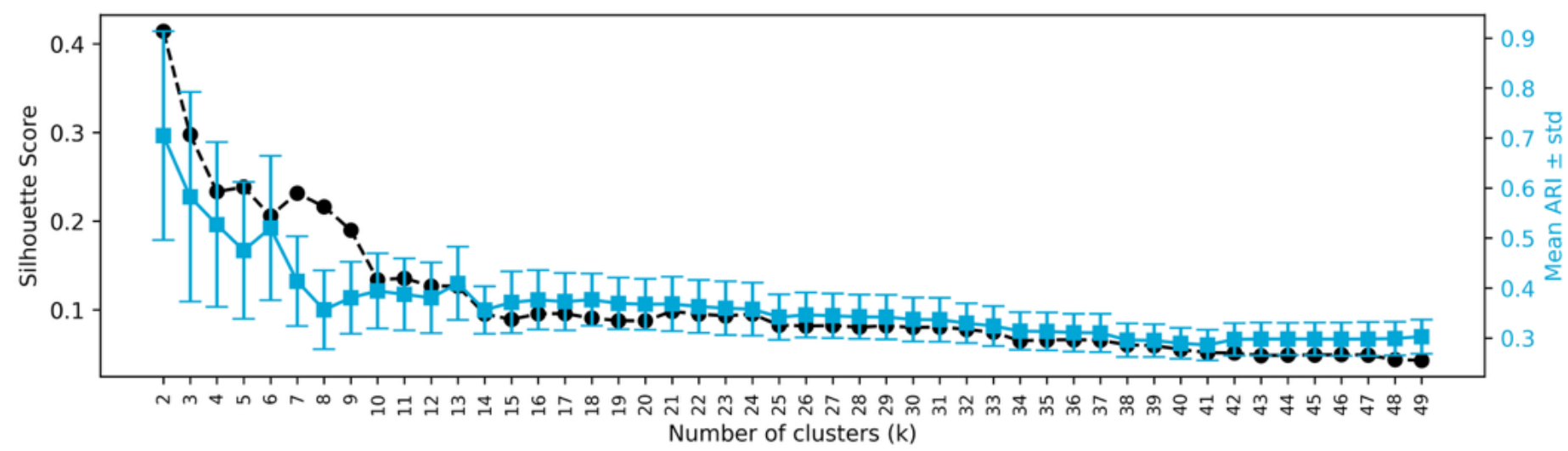

Chosen k = 6 according to the silhouette score and cluster stability (the Adjusted Rand Index, ARI), and after the analysis of the distributions of features used in clustering.

References

Arthur D, Vassilvitskii S (2007) K-means++: the advantages of careful seeding. In Proceedings of the eighteenth annual ACM-SIAM symposium on Discrete algorithms (SODA '07). Society for Industrial and Applied Mathematics, USA, 1027–1035.

Camus P, Mendez FJ, Medina R, Cofiño AS (2011) Analysis of clustering and selection algorithms for the study of multivariate wave climate. Coastal Engineering 58:453–462. https://doi.org/10.1016/j.coastaleng.2011.02.003

Kaufman L, et al (1990) Partitioning around Medoids (Program PAM). In: Kaufman, L. and Rousseeuw, P., Eds., Finding Groups in Data: An Introduction to Cluster Analysis, John Wiley, New York, 68-125.

Table S1 Data metadata

Station number	Station name	Country	Latitude	Longitude	Sampling interval	Data start time	Data end time	Data source[1]	Data source switch time[2]
**1.**	Maloy	Norway	61.933333	5.116667	10	1.1.2008 0:00	1.1.2025 0:00	Kartverket	
**2.**	Tregde	Norway	58	7.566667	10	1.1.2008 0:00	1.1.2025 0:00	Kartverket	
**3.**	Hirtshals	Denmark	57.6	9.97	10	3.8.2008 12:20	1.1.2021 0:00	IOC SLSMF	
**4.**	Delfzijl*	Netherlands	53.3266	6.9329	10	1.1.1993 0:00	31.12.2024 23:00	GESLA & RWS	31.12.2017 23:00
**5.**	Huibertgat*	Netherlands	53.573748	6.398433	10	1.1.1993 0:00	31.12.2024 23:00	GESLA & RWS	31.12.2017 23:00
**6.**	Terschelling*	Netherlands	53.4278	5.3328	10	1.1.1993 0:00	31.12.2024 23:00	GESLA & RWS	31.12.2017 23:00
**7.**	Harlingen*	Netherlands	53.17556	5.41097	10	1.1.1993 0:00	31.12.2024 23:00	GESLA & RWS	31.12.2017 23:00
**8.**	Den Helder*	Netherlands	52.9636	4.7434	10	1.1.1993 0:00	31.12.2024 23:00	GESLA & RWS	31.12.2017 23:00
**9.**	IJmuiden Buitenhaven*	Netherlands	52.462323	4.554823	10	1.1.1993 0:00	30.6.2024 22:50	GESLA & RWS	31.12.2017 22:50
**10.**	Scheveningen*	Netherlands	52.09893226	4.263173332	10	1.1.1993 0:00	31.12.2024 23:00	GESLA & RWS	31.12.2017 23:00
**11.**	Hoek van Holland*	Netherlands	51.97747	4.11949	10	1.1.1993 0:00	31.12.2024 23:00	GESLA & RWS	31.12.2017 22:50
**12.**	Brouwershavensche Gat*	Netherlands	51.750754	3.811483	10	1.1.1993 0:00	30.6.2024 22:50	GESLA & RWS	31.12.2017 23:00
**13.**	Europlatform*	Netherlands	51.99769	3.274687	10	30.6.2001 23:00	31.12.2024 23:00	GESLA & RWS	31.12.2017 23:00
**14.**	Terneuzen*	Netherlands	51.33620086	3.819502676	10	1.1.1993 0:00	1.1.2021 0:00	GESLA & IOC SLSMF	11.1.2017 20:10
**15.**	Vlissingen*	Netherlands	51.44285825	3.597008647	10	1.1.1993 0:00	31.12.2024 23:00	GESLA & RWS	31.12.2017 23:00
**16.**	Breskens	Netherlands	51.4014	3.5477	10	9.3.2017 9:10	1.1.2021 0:00	IOC SLSMF	
**17.**	Vlakte	Netherlands	51.50361684	3.241661685	10	4.8.2014 0:00	1.1.2021 0:00	GESLA & IOC SLSMF	11.1.2017 19:50
**18.**	Ostend	Belgium	51.2330556	2.9205556	5	6.9.2013 0:00	1.1.2021 0:00	IOC SLSMF	
**19.**	Dover*	UK	51.11	1.32	15	1.1.1993 0:00	1.1.2021 0:00	GESLA & IOC SLSMF	3.9.2019 20:15
**20.**	Deal Pier (10 min)	UK	51.22379	1.40926	10	1.1.2006 0:10	05.10.2012 23:50	GESLA	
**21.**	Deal Pier (1 min)	UK	51.22379	1.40926	1	5.10.2012 9:09	21.12.2020 21:27	IOC SLSMF	

**22.**	Sheerness	UK	51.45	0.74	15	1.1.1993 0:15	1.1.2021 0:00	GESLA & IOC SLSMF	3.9.2019 20:15
**23.**	Lowestoft*	UK	52.47	1.75	15	1.1.1993 0:15	1.1.2021 0:00	GESLA & IOC SLSMF	3.9.2019 20:00
**24.**	Immingham*	UK	53.63	-0.19	15	1.1.1993 0:15	1.1.2021 0:00	GESLA & IOC SLSMF	3.9.2019 20:15
**25.**	Whitby*	UK	54.49	-0.61	15	1.1.1993 0:00	1.1.2021 0:00	GESLA & IOC SLSMF	3.9.2019 20:15
**26.**	Leith	UK	55.99	-3.18	15	1.1.1993 0:00	1.1.2021 0:00	GESLA & IOC SLSMF	3.9.2019 20:15
**27.**	Wick Harbour*	UK	58.44	-3.09	15	1.1.1993 0:15	1.1.2021 0:00	GESLA & IOC SLSMF	3.9.2019 20:15
**28.**	Lerwick (1 min)	UK	60.155316	-1.145192	1	6.3.2009 17:31	24.2.2018 19:32	IOC SLSMF	
**29.**	Lerwick (15 min)	UK	60.155316	-1.145192	15	1.1.1993 0:00	1.1.2021 0:00	GESLA & IOC SLSMF	26.4.2012 9:45

[1] In cases where multiple data sources were used, these are listed in the table in the order in which they were used.
[2] **Data source switch time** refers to the moment when the data source was changed from one to another.
*Stations used for spatial clustering.

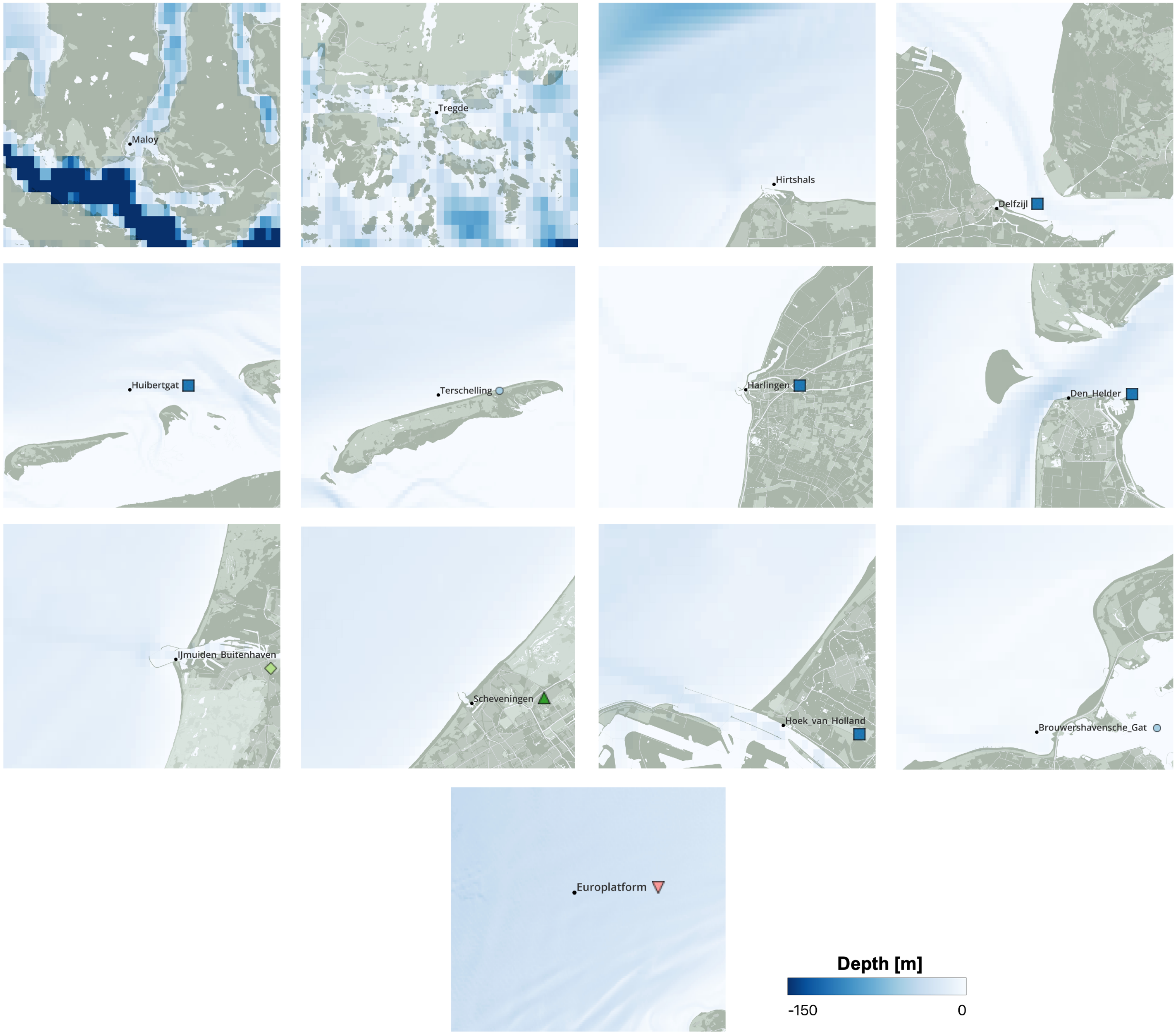
Maloy
Tregde
Hirtshals
Delfzijl
Huibertgat
Terschelling
Harlingen
Den_Helder
IJmuiden_Buitenhaven
Scheveningen
Hoek_van_Holland
Brouwershavensche_Gat
Europlatform
Depth [m]
-150
0

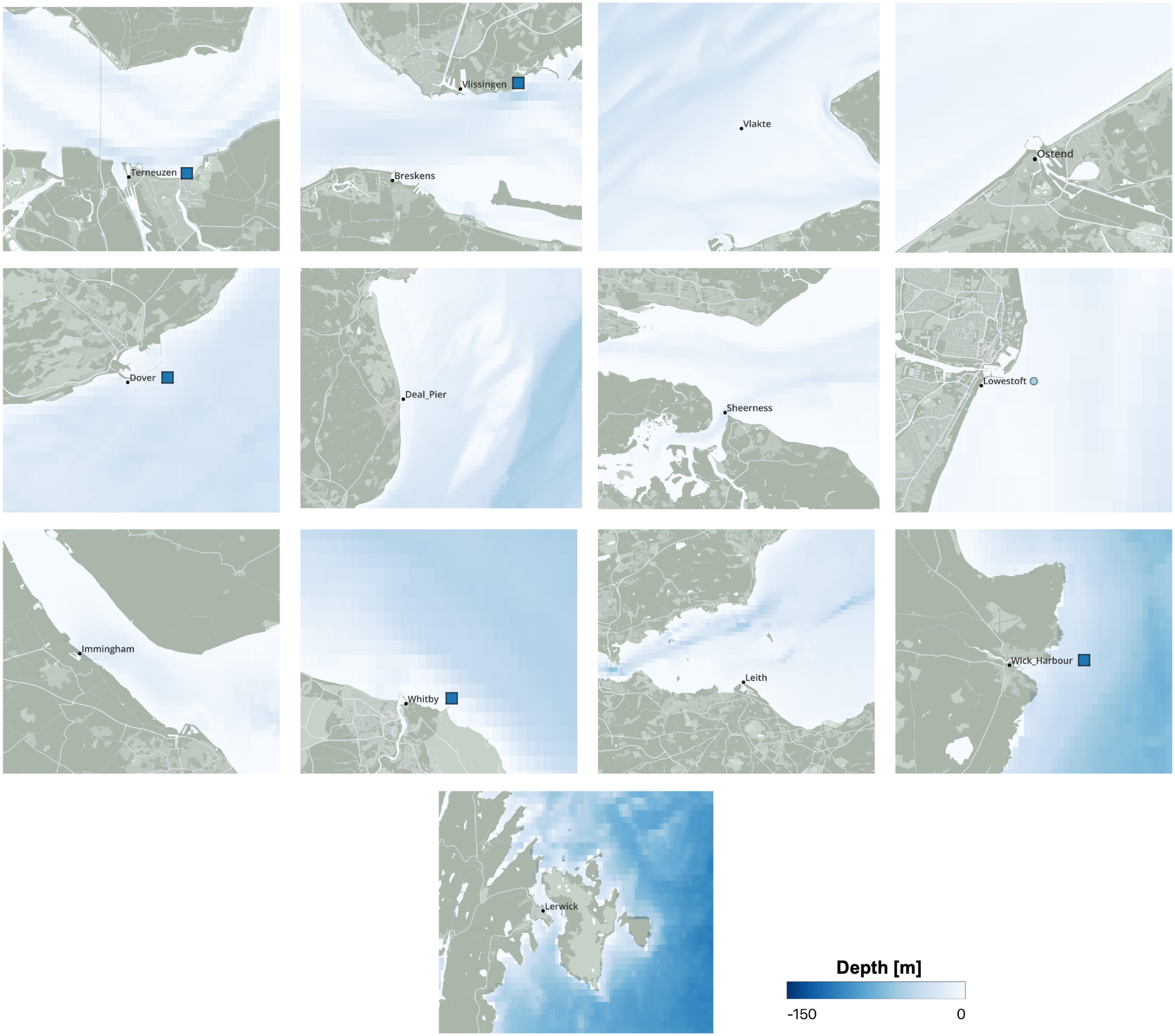
Terneuzen
Vlissingen
Breskens
Vlakte
Ostend
Dover
Deal_Pier
Sheerness
Lowestoft
Immingham
Whitby
Leith
Wick_Harbour
Lerwick
Depth [m]
-150
0
